# Supplementary material for: Modeling functional cell types in spike train data
Source: PLoS Comput Biol. 2023 Oct 12;19(10):e1011509. doi: 10.1371/journal.pcbi.1011509 (PMC10569560; doi:10.1371/journal.pcbi.1011509)
Supplement: S1 Text — Additional details regarding methods and results, as well as supplementary analyses. (PDF) [file pcbi.1011509.s001.pdf]

Supplementary Information for the article: Modeling functional cell types in spike train data

A Extended EM Methods

Here we provide a more detailed account of how our adapted EM algorithm fits our hierarchical generative model (11) to data.

A.1 E-Step

Our expectation (E) step consists of finding the $Z_{i,k}, \boldsymbol{m}_{i,k}, c_{i,k}$ that best approximate the posterior distribution over the latent variables, $Q_i(k, \boldsymbol{\beta})$ (17).

For fixed i and k, we first set $\boldsymbol{m}_{i,k}$ so that the modes of the left and right hand sides of (14) are equal. To do this, we apply the trust-region Newton-conjugate-gradient algorithm to solve the convex optimization problem

$$\boldsymbol{m}_{i,k} = \arg\max_{\boldsymbol{\beta}} \log P_{\text{joint}}(k, \boldsymbol{\beta}, \mathbf{y}_i | \mathbf{x}_i; \hat{\Omega}_K). \tag{A}$$

Next, so that the Hessians of the logs of the left and right hand sides of (14) are equal, we compute

$$\tilde{c}_{i,k}^{-1} = -\nabla^2_{\boldsymbol{\beta}} \log P_{\text{joint}}(k, \boldsymbol{\beta}, \mathbf{y}_i | \mathbf{x}_i; \hat{\Omega}_K)\Big|_{\boldsymbol{\beta}=\boldsymbol{m}_{i,k}}. \tag{B}$$

The Hessian of a GLM log-likelihood function is often used during optimization (see [1] 9.3.2 for details), and we make use of the `statsmodels` package to efficiently compute it. We further approximate $\tilde{c}_{i,k}^{-1}$ by retaining only its diagonal elements, i.e. $c_{i,k}^{-1} = \text{diag}(\tilde{c}_{i,k}^{-1})$.

Finally, noting that $f(\boldsymbol{\beta}; \boldsymbol{m}_{i,k}, c_{i,k})\Big|_{\boldsymbol{\beta}=\boldsymbol{m}_{i,k}} = \frac{1}{\sqrt{(2\pi)^{T^{\text{stim}}+T^{\text{self}}+1}|c_{i,k}|}}$, we take

$$Z_{i,k} = P_{\text{joint}}(k, \boldsymbol{\beta}, \mathbf{y}_i | \mathbf{x}_i; \hat{\Omega}_K)\Big|_{\boldsymbol{\beta}=\boldsymbol{m}_{i,k}} \sqrt{(2\pi)^{T^{\text{stim}}+T^{\text{self}}+1}|c_{i,k}|}, \tag{C}$$

where $|c_{i,k}|$ denotes the determinant, so that the left and right hand sides of (14) are equal when $\boldsymbol{\beta} = \boldsymbol{m}_{i,k}$. Collectively, (A), (B), and (C) are known as the Laplace approximation (see [1] 8.4.1).

This concludes the set of operations needed to perform the E-step.

A.2 M-Step

The maximization (M) step consists of finding Ω_K that maximizes the lower bound on the log-likelihood, (18). In light of (8), the bound being maximized in the M-step factors as follows:

$$\sum_{i=1}^{N}\sum_{k=1}^{K}\int \tilde{Z}_{i,k} f(\boldsymbol{\beta}; \boldsymbol{m}_{i,k}, c_{i,k}) \log P_{\text{joint}}(k, \boldsymbol{\beta}, \mathbf{y}_i | \mathbf{x}_i; \Omega_K) d\boldsymbol{\beta}$$

$$= \sum_{i=1}^{N}\sum_{k=1}^{K}\int \tilde{Z}_{i,k} f(\boldsymbol{\beta}; \boldsymbol{m}_{i,k}, c_{i,k}) [\log P_{\text{SC}}(\mathbf{y}_i | \mathbf{x}_i, \boldsymbol{\beta}) + \log \pi_k + \log f(\boldsymbol{\beta}; \boldsymbol{\mu}_k, \Sigma_k)] d\boldsymbol{\beta}.$$

Absorbing into C terms that do not depend on the optimization variables Ω_K, this equal

$$\sum_{i=1}^{N}\sum_{k=1}^{K}\int \tilde{Z}_{i,k} f(\boldsymbol{\beta}; \boldsymbol{m}_{i,k}, c_{i,k}) [\log \pi_k + \log f(\boldsymbol{\beta}; \boldsymbol{\mu}_k, \Sigma_k)] d\boldsymbol{\beta} + C.$$

Rearranging the sums and integrals, this equals

$$\sum_{k=1}^{K}\int [\sum_{i=1}^{N} \tilde{Z}_{i,k} f(\boldsymbol{\beta}; \boldsymbol{m}_{i,k}, c_{i,k})] [\log \pi_k + \log f(\boldsymbol{\beta}; \boldsymbol{\mu}_k, \Sigma_k)] d\boldsymbol{\beta} + C.$$

Distributing the product and using $\int f(\boldsymbol{\beta}; \boldsymbol{m}_{i,k}, c_{i,k}) d\boldsymbol{\beta} = 1$ to simplify the first term, th

$$\sum_{k=1}^{K}[\sum_{i=1}^{N} \tilde{Z}_{i,k}] \log \pi_k + \sum_{k=1}^{K}\int [\sum_{i=1}^{N} \tilde{Z}_{i,k} f(\boldsymbol{\beta}; \boldsymbol{m}_{i,k}, c_{i,k})] \log f(\boldsymbol{\beta}; \boldsymbol{\mu}_k, \Sigma_k) d\boldsymbol{\beta} + C.$$

We maximize the first term in (D), $\sum_{k=1}^{K}[\sum_{i=1}^{N} \tilde{Z}_{i,k}] \log \pi_k$, with respect to $\pi_1, ..., \pi_K$, subject to $\pi_k \geq 0, k = 1, ..., K$ and $\sum_{k=1}^{K} \pi_k = 1$. Algebraic manipulation reveals that

$$\hat{\pi}_k = \frac{1}{N}\sum_{i=1}^{N} \tilde{Z}_{i,k}. \tag{E}$$

Each summand in the second term in (D), $\int [\sum_{i=1}^{N} \tilde{Z}_{i,k} f(\boldsymbol{\beta}; \boldsymbol{m}_{i,k}, c_{i,k})] \log f(\boldsymbol{\beta}; \boldsymbol{\mu}_k, \Sigma_k) d\boldsymbol{\beta}$, is maximized independently with respect to $\boldsymbol{\mu}_k$ and Σ_k. We use the probability density function for a multivariate Gaussian to rewrite this term as

$$\int \left[\sum_{i=1}^{N} \tilde{Z}_{i,k} f(\boldsymbol{\beta}; \boldsymbol{m}_{i,k}, c_{i,k})\right] \left[-\frac{(\boldsymbol{\beta} - \boldsymbol{\mu}_k)^\top \Sigma_k^{-1} (\boldsymbol{\beta} - \boldsymbol{\mu}_k) - (T^{\text{stim}} + T^{\text{self}} + 1) \log(2\pi) + \log(|\Sigma_k^{-1}}{2}\right. \tag{F}$$

Differentiating (F) with respect to $\boldsymbol{\mu}_k$ reveals that

$$\begin{aligned} 0 &= \int \left[\sum_{i=1}^{N} \tilde{Z}_{i,k} f(\boldsymbol{\beta}; \boldsymbol{m}_{i,k}, c_{i,k})\right] \Sigma_k^{-1} (\boldsymbol{\beta} - \boldsymbol{\mu}_k) d\boldsymbol{\beta} \\ &= \sum_{i=1}^{N} \tilde{Z}_{i,k} \Sigma_k^{-1} \left[\int f(\boldsymbol{\beta}; \boldsymbol{m}_{i,k}, c_{i,k}) \boldsymbol{\beta} d\boldsymbol{\beta} - \int f(\boldsymbol{\beta}; \boldsymbol{m}_{i,k}, c_{i,k}) \boldsymbol{\mu}_k d\boldsymbol{\beta}\right] \\ &= \sum_{i=1}^{N} \tilde{Z}_{i,k} \Sigma_k^{-1} \left[\boldsymbol{m}_{i,k} - \boldsymbol{\mu}_k\right]. \end{aligned}$$

This gives us

$$\hat{\boldsymbol{\mu}}_k = \frac{\sum_{i=1}^{N} \tilde{Z}_{i,k} \boldsymbol{m}_{i,k}}{\sum_{i=1}^{N} \tilde{Z}_{i,k}}. \tag{G}$$

To maximize (F) with respect to Σ_k, we note that it is concave in Σ_k^{-1}. We can then solve for Σ_k by setting the derivative with respect to Σ_k^{-1} equal to zero:

$$0 = \int \sum_{i=1}^{N} \tilde{Z}_{i,k} f(\boldsymbol{\beta}; \boldsymbol{m}_{i,k}, c_{i,k}) \left[-\frac{1}{2} (\boldsymbol{\beta} - \boldsymbol{\mu}_k)(\boldsymbol{\beta} - \boldsymbol{\mu}_k)^\top + \frac{1}{2} \Sigma_k \right] d\boldsymbol{\beta},$$

making use of the fact that $\frac{d}{dX} \log(|X|) = (X^{-1})^\top$, and that $\Sigma_k = \Sigma_k^\top$. That is,

$$\Sigma_k = \frac{\sum_{i=1}^{N} \tilde{Z}_{i,k} \mathbb{E}\left[(\boldsymbol{\beta} - \boldsymbol{\mu}_k)(\boldsymbol{\beta} - \boldsymbol{\mu}_k)^\top \right]}{\sum_{i=1}^{N} \tilde{Z}_{i,k}},$$

where the expectation is with respect to $\boldsymbol{\beta} \sim \mathcal{N}(\boldsymbol{m}_{i,k}, c_{i,k})$. We can decompose this ex

$$\begin{aligned}
& \mathbb{E}_{\boldsymbol{\beta} \sim \mathcal{N}(\boldsymbol{m}_{i,k}, c_{i,k})} \left[(\boldsymbol{\beta} - \boldsymbol{\mu}_k)(\boldsymbol{\beta} - \boldsymbol{\mu}_k)^\top \right] \\
= {} & \mathbb{E}_{\boldsymbol{\beta} \sim \mathcal{N}(\boldsymbol{m}_{i,k}, c_{i,k})} \left[(\boldsymbol{\beta} - \boldsymbol{m}_{i,k} + \boldsymbol{m}_{i,k} - \boldsymbol{\mu}_k)(\boldsymbol{\beta} - \boldsymbol{m}_{i,k} + \boldsymbol{m}_{i,k} - \boldsymbol{\mu}_k)^\top \right] \\
= {} & \mathbb{E}_{\boldsymbol{\beta} \sim \mathcal{N}(\boldsymbol{m}_{i,k}, c_{i,k})} \left[(\boldsymbol{\beta} - \boldsymbol{m}_{i,k})(\boldsymbol{\beta} - \boldsymbol{m}_{i,k})^\top + (\boldsymbol{m}_{i,k} - \boldsymbol{\mu}_k)(\boldsymbol{m}_{i,k} - \boldsymbol{\mu}_k)^\top \right. \\
& \left. + (\boldsymbol{\beta} - \boldsymbol{m}_{i,k})(\boldsymbol{m}_{i,k} - \boldsymbol{\mu}_k)^\top + (\boldsymbol{m}_{i,k} - \boldsymbol{\mu}_k)(\boldsymbol{\beta} - \boldsymbol{m}_{i,k})^\top \right] \\
= {} & c_{i,k} + (\boldsymbol{m}_{i,k} - \boldsymbol{\mu}_k)(\boldsymbol{m}_{i,k} - \boldsymbol{\mu}_k)^\top.
\end{aligned}$$

Plugging this expression back in for the expectation, we have

$$\begin{aligned}
\Sigma_k &= \frac{\sum_{i=1}^{N} \tilde{Z}_{i,k} (c_{i,k} + \boldsymbol{m}_{i,k} \boldsymbol{m}_{i,k}^\top + \boldsymbol{\mu}_k \boldsymbol{\mu}_k^\top - \boldsymbol{m}_{i,k} \boldsymbol{\mu}_k^\top - \boldsymbol{\mu}_k \boldsymbol{m}_{i,k}^\top)}{\sum_{i=1}^{N} \tilde{Z}_{i,k}} \\
&= \frac{\sum_{i=1}^{N} \tilde{Z}_{i,k} (c_{i,k} + \boldsymbol{m}_{i,k} \boldsymbol{m}_{i,k}^\top - \boldsymbol{\mu}_k \boldsymbol{\mu}_k^\top)}{\sum_{i=1}^{N} \tilde{Z}_{i,k}},
\end{aligned}$$

where in the last step we make use of (G) to simplify the last two terms in the numerator. Finally, subjecting this expression to the constraint that Σ_k must be diagonal, we have:

$$\hat{\Sigma}_k = \frac{\sum_{i=1}^{N} \tilde{Z}_{i,k} \mathrm{diag}(c_{i,k} + \boldsymbol{m}_{i,k} \boldsymbol{m}_{i,k}^\top - \hat{\boldsymbol{\mu}}_k \hat{\boldsymbol{\mu}}_k^\top)}{\sum_{i=1}^{N} \tilde{Z}_{i,k}}. \tag{H}$$

In the main manuscript, the solutions (G) and (H) are applied only to $\boldsymbol{\mu}^{\text{self}}, \Sigma^{\text{self}}$, as the other components are held fixed during EM.

A.3 Computational complexity, runtime information, and job load

Each iteration of the E step must perform $N \times K$ convex gradient descent sub-optimizations, for every neuron and every cluster, through a space of $T^{\text{stim}} + T^{\text{self}} + 1$ parameters on T data points. This operation is the most costly, as the M step is a simple calculation.

In practice, using a warm start for these convex gradient descent sub-optimizations, where the results of last iteration's sub-optimizations are used to initialize those of this iteration, dramatically reduces the runtime of subsequent iterations. Concretely, for Case B applied to $T = 20,000$ time bins of Allen Cell Types Database data, each sub-optimization took about 40ms after the warm start had kicked in.

The number of iterations necessary for the EM algorithm to converge is difficult to predict theoretically. In practice, the relative change in loss ($\frac{\text{Loss(iter}-1)-\text{Loss(iter)}}{\text{Loss(iter)}}$, where $\text{Loss} \equiv \sum_{i=1}^{N} \text{LL}_i$, with LL_i defined in Section 2.4) reached 10^{-7} in at most 55 iterations for $K = 20$ applied to all $N = 634$ Allen Cell Types Database neurons. For smaller values of K, fewer iterations are necessary.

The total runtime for a single initialization of the EM algorithm on Allen Cell Types Database data with $K = 20$ was around 5 hours or less for Case A (for a single choice of λ^{stim}) and 21 hours or less for Case B. All optimizations were performed on individual compute nodes with at least 32 cores and 256G of memory. We performed separate optimizations in parallel on separate nodes to produce different hyperparameter combinations and random initializations. For Case A with the Allen Cell Types Database data, we varied $K \in \{1, ..., 20\}$, $\lambda^{\text{stim}} \in \{10^{-9}, 10^{-8\frac{2}{3}}, ..., 10^{-1}\}$, and ran the EM algorithm for 20 different random initializations, yielding a total of $20 \times 26 \times 20 = 14000$ separate runs.

B Alternative Model: all parameters depend on cell-type

Here, we consider an alternate formulation of both sequential and simultaneous methods, where instead of just $\boldsymbol{\beta}_i^{\text{self}}$ being related to cell-type, as considered in the main manuscript, we consider the case where all of $\boldsymbol{\beta}_i$ is related to cell-type. We call the former, in main, case A, and the latter, detailed here, case B.

We note here that providing analysis of the relative benefits of case A and case B is not a major objective of this work. Rather, we seek to demonstrate only that there are options for how one chooses to define a hierarchical model within our general framework, and each may have its own benefits and drawbacks, depending on the line of inquiry. As the rest of this section will elaborate, this option is equally present for both the simultaneous and sequential methods.

We focus on case A in the main primarily because it provides a simpler model than B: it selects fewer clusters, lower within-cluster variances (Σ_k), and by its definition is a lower dimensional model. All of these factors give case A an advantage over case B in terms of interpretability. Additionally, case A performs better than B in terms of model selection on simulated data (compare Figs 3, HA, and HB with Figs B, HC, and HD). Finally, the shared variables in case A, the self-interaction filters, are more strongly associated with intrinsic neural dynamics, whereas, at least in the IVSCC dataset, the stimulus-filters may depend more on other factors, such as the impedance of the electrode-neuron interface used to administer the stimulus. This, along with the fact that case A is lower dimensional, may account for any relatively better performance by case A. However we also acknowledge that there are cases where case B may be preferable, such as when one specifically wishes to distinguish the stimulus processing properties of neurons.

B.1 Alternative Methods

Below we detail how the sequential and simultaneous methods change for case B. For both, the main difference is that instead of fitting a cluster model with means $\boldsymbol{\mu}_k^{\text{self}}$ and covariance matrices Σ_k^{self} that describes the distribution of $\boldsymbol{\beta}_i^{\text{self}}$, they now fit one with means $\boldsymbol{\mu}_k$ and covariance matrices Σ_k that describes the distribution of $\boldsymbol{\beta}_i$. For convenience, we will refer to the appropriate components of $\boldsymbol{\mu}_k$ and Σ_k using superscripts, e.g. $\boldsymbol{\mu}_k^{\text{stim}}$, just as we do with $\boldsymbol{\beta}_i$.

B.1.1 Sequential Method

Because, in the sequential method, the fitting of single cell models does not depend on cell-types, that first stage is identical between cases A and B. For the second stage, clustering, the only difference is that GMM clustering is performed on all of $\hat{\boldsymbol{\beta}}_i$, instead of just $\hat{\boldsymbol{\beta}}_i^{\text{self}}$:

$$\hat{\Omega}_K \leftarrow \arg\max_{\Omega_K} \sum_{i=1}^{N} \log \sum_{k=1}^{K} \pi_k f(\hat{\boldsymbol{\beta}}_i; \boldsymbol{\mu}_k, \Sigma_k) \tag{I}$$

$$\hat{k}_i \leftarrow \arg\max_k \hat{\pi}_k f(\hat{\boldsymbol{\beta}}_i; \hat{\boldsymbol{\mu}}_k, \hat{\Sigma}_k),\ i = 1, ..., N \tag{J}$$

Algorithm 1 is thus unchanged except for changing the last two lines accordingly. The computation of the BIC also changes accordingly:

$$BIC \equiv \sum_{i=1}^{N} \log \left[\sum_{k=1}^{K} \hat{\pi}_k f(\hat{\boldsymbol{\beta}}_i; \hat{\boldsymbol{\mu}}_k, \hat{\Sigma}_k) \right] - \frac{K - 1 + 2K\dim(\boldsymbol{\beta})}{2} \log(N) \tag{K}$$

B.1.2 Simultaneous Method

For the simultaneous method, case B is much simpler, as all parameters of the single cell models use the learned cluster structure as priors, so there is no longer a need for ℓ_2 regularization.

The joint probability of the data and parameters simplifies instead to:

$$P_{\text{joint}}(k, \boldsymbol{\beta}, \mathbf{y}_i | \mathbf{x}_i; \Omega_K) \propto P_{SC}(\mathbf{y}_i | \mathbf{x}_i; \boldsymbol{\beta}) f(\boldsymbol{\beta}_i^{\text{stim}}; \boldsymbol{\mu}_k^{\text{stim}}, \Sigma_k^{\text{stim}}) f(\boldsymbol{\beta}_i^{\text{self}}; \boldsymbol{\mu}_k^{\text{self}}, \Sigma_k^{\text{self}}) f(\beta_i^0; \mu_k^0, \Sigma_k^0) \pi_k. \tag{L}$$

With this adapted P_{joint} the results derived in the main manuscript, i.e. (12)-(21), all apply to case B, without making the exceptions for constrained cluster means and covariances, as was done for G,H. Algorithm 2 is thus unchanged except that all of $\boldsymbol{\mu}_k$ and Σ_k are updated in the M-step instead of just $\boldsymbol{\mu}_k^{\text{self}}$ and Σ_k^{self}. For (21), in case B we have $\text{dof}(\hat{\Omega}_K) = K * (2 * (T^{\text{self}} + T^{\text{stim}} + 1) + 1) - 1$, reflecting the change in number of free parameters.

B.2 Results on Simulated Data

Here we show the equivalent results to those presented in Figs 2 and 3, but for the alternative model where all parameters depend on cell-type. For these results, the simulated dataset was sampled from GLMs whose true $\boldsymbol{\mu}^{\text{stim}}$ and μ^0 were clustered by cell-type, in addition to $\boldsymbol{\mu}^{\text{self}}$ (Fig AA and AB).

Fig AC-AF show very similar results for parameter recovery to those in Fig 2, the only salient difference being that the recovery of $\boldsymbol{\beta}_i^{\text{stim}}$ is also much better in the simultaneous method, and for lower σ. This is to be expected now that the true values of these parameters depend on sigma, and their estimates in the simultaneous method make use of cell-type specific priors.

Model selection, however, has markedly worse performance with the all-parameters-shared model, and we can no longer say that the simultaneous method is better (Fig B). Since the $\boldsymbol{\mu}^{\text{self}}$ are the same in all cases we consider, we can interpret this result by saying that for our choice of Ω_K, the additional differences between clusters in $\boldsymbol{\mu}^{\text{stim}}$ and μ^0 do not make it easier enough to separate the clusters to overcome the increased complexity penalty in BIC with increased $\text{dof}(\hat{\Omega}_K)$.

B.3 Results on Allen Cell Types Database Data

Here we show results like those shown in Figs 4,5, and 6, but using the alternate model where all parameters are cell-type-dependent (case B).

In Figs C, D, and E, we compare between simultaneous and sequential methods the discovered cluster structure, the generalization performance of fitted models, and the link between cluster labels and metadata, respectively, resulting from fitting to the Allen Cell Types Database data.

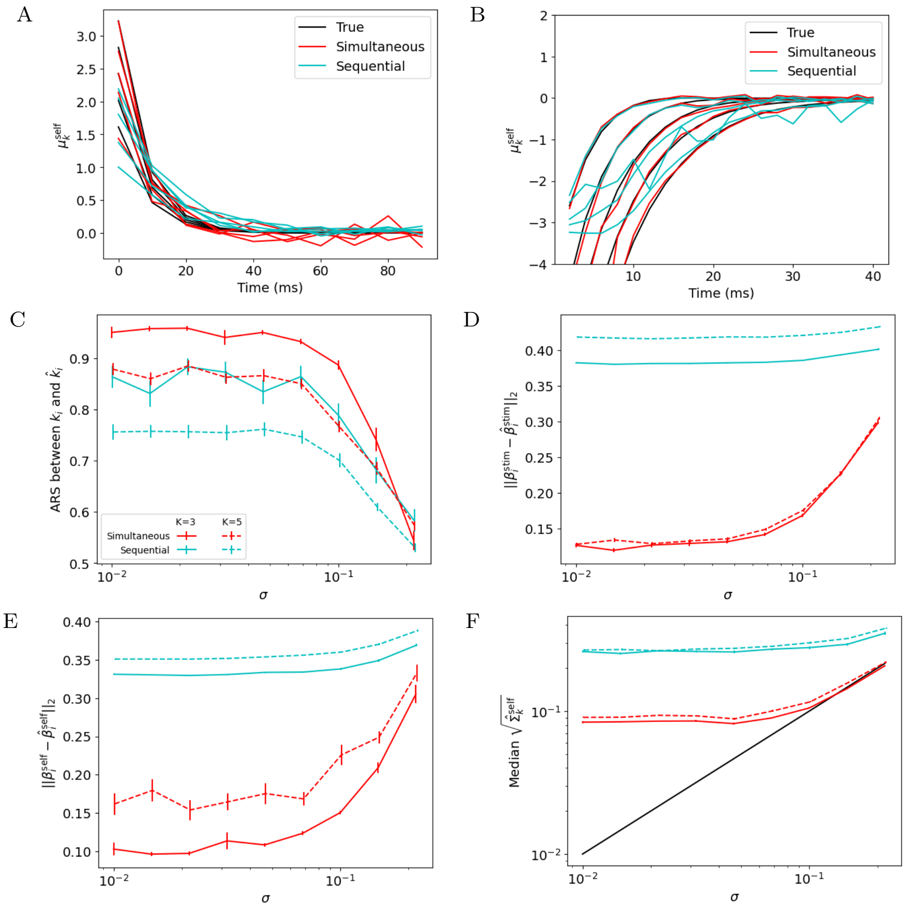

Fig A: **Performance of simultaneous and sequential methods with all parameters shared on data simulated from a fixed cluster structure.**
A, B: The true cluster means $\boldsymbol{\mu}_k^{\text{stim}}, \boldsymbol{\mu}_k^{\text{self}}$ used to generate simulated datasets and those estimated by the sequential and simultaneous methods, fit with the correct $K = 5$.
C-F: same plots as Fig 2, but with the alternative model. In all metrics and conditions except ARS with $K = 3$ and the two highest values of σ, the simultaneous method is significantly better.

To assess how similar the cluster assignments discovered by this alternate method are to those presented in main, we show the confusion matrix in Fig FA for the simultaneous method, and in Fig FB for the sequential method. For completeness, we also show the other 4 pairs of comparisons between different clusterings. In each panel, the rows and columns are ordered independently to maximize the sum along the main diagonal. This is achieved using the Hungarian algorithm, implemented in `scipy.optimize.linear_sum_assignment`. Afterwards the values are normalized by the geometric mean of the row- and column-sums, so that the plot is not dominated by the larger clusters.

All four combinations of method and case produce very different clusterings, but the two results of the sequential method produce the most similar results. This might be expected from the fact that these use identical estimates of self-interaction filters $\hat{\boldsymbol{\beta}}_i^{\text{self}}$, which are used in both clusterings. The fact that there is such good agreement may be taken to mean that the other GLM parameters do not convey much useful information

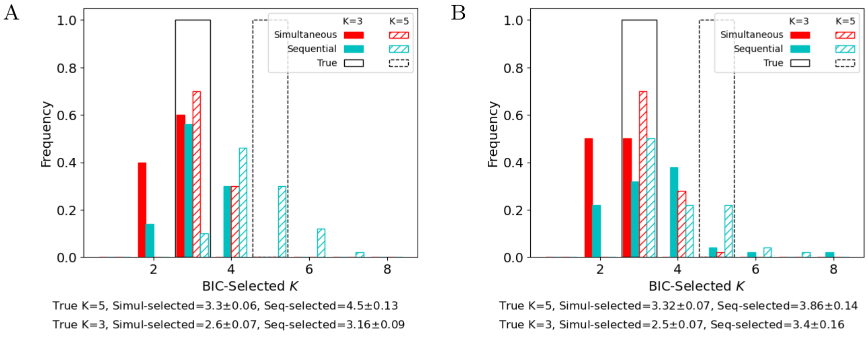

Fig B: **Model selection of $\hat{K}$ using BIC with the alternative model.** Frequencies of $\hat{K}$ estimated via BIC over 50 simulated datasets with the same μ_k as in Fig A, and $\sigma = 10^{-2}$ (A) or $10^{-5/6}$ (B), the maximum value that does not result in degenerate simulations. Black lines indicate true K. Summary below plots gives the mean $\pm$ SEM of estimated $\hat{K}$ across the 50 datasets for each case and each method.

about cell type, as the heavy overlap between clusters in the stimulus filter dimensions would suggest (see Fig CF).

The greater change is seen for the simultaneous method, both in the clustering assignments (see Fig FA), and in the increased spread of clusters along the self-interaction filter dimension ($\sqrt{\Sigma_k^{\text{self}}}$, shaded bars in Fig CB). The latter may be explained by the fact that here we are clustering in a higher-dimensional space, and therefore clusters will generally spread wider along an existing dimension to account for the extra variability added in the new dimensions. This explanation presumes that the new dimensions provide new variability that is different enough from that in the existing dimensions to meaningfully change cluster assignments, as is indeed the case (see Fig FA). Why the variability in stimulus filter dimensions is different from in self-interaction filter dimensions is a difficult question to answer rigorously, but we propose that the self-interaction filters may truly be more purely related to intrinsic dynamics, whereas the stimulus filter may also be accounting for elements of the electrode-neuron interface. These elements may include electrical properties of the interface and the electrode placement in relation to the morphology of the neuron.

The fact that a similar pattern does not emerge for the sequential method may be a result of the fact that the discovered clusters overlap heavily in the stimulus filter dimensions, as shown in Fig CF, whereas there are distinct differences between clusters for the simultaneous method (Fig CE). This pattern is expected from our general observation that the simultaneous method can use borrowed strength between neurons to collapse clusters and increase their separation. As such, the addition of stimulus filters to the clustering does not change the cluster assignments very much (see Fig FB), and thus the positions of the clusters in the self-interaction filter dimensions do not change substantially.

C Additional Details for Figs 5 and D

The following descriptions apply to both Figs 5 and D, except for panels B, D, and F, which do not exist in Fig D, as these analyses have not been performed. The results in Fig 5 are from models where only the self-interaction filters are shared ("Case A" - see Sections 2.2 and 2.3), whereas those in Fig D are from models where all parameters are shared ("Case B" - see Sections B.1.1 and B.1.2).

To generate panels A, C, and E, we split the neurons into four folds, and applied

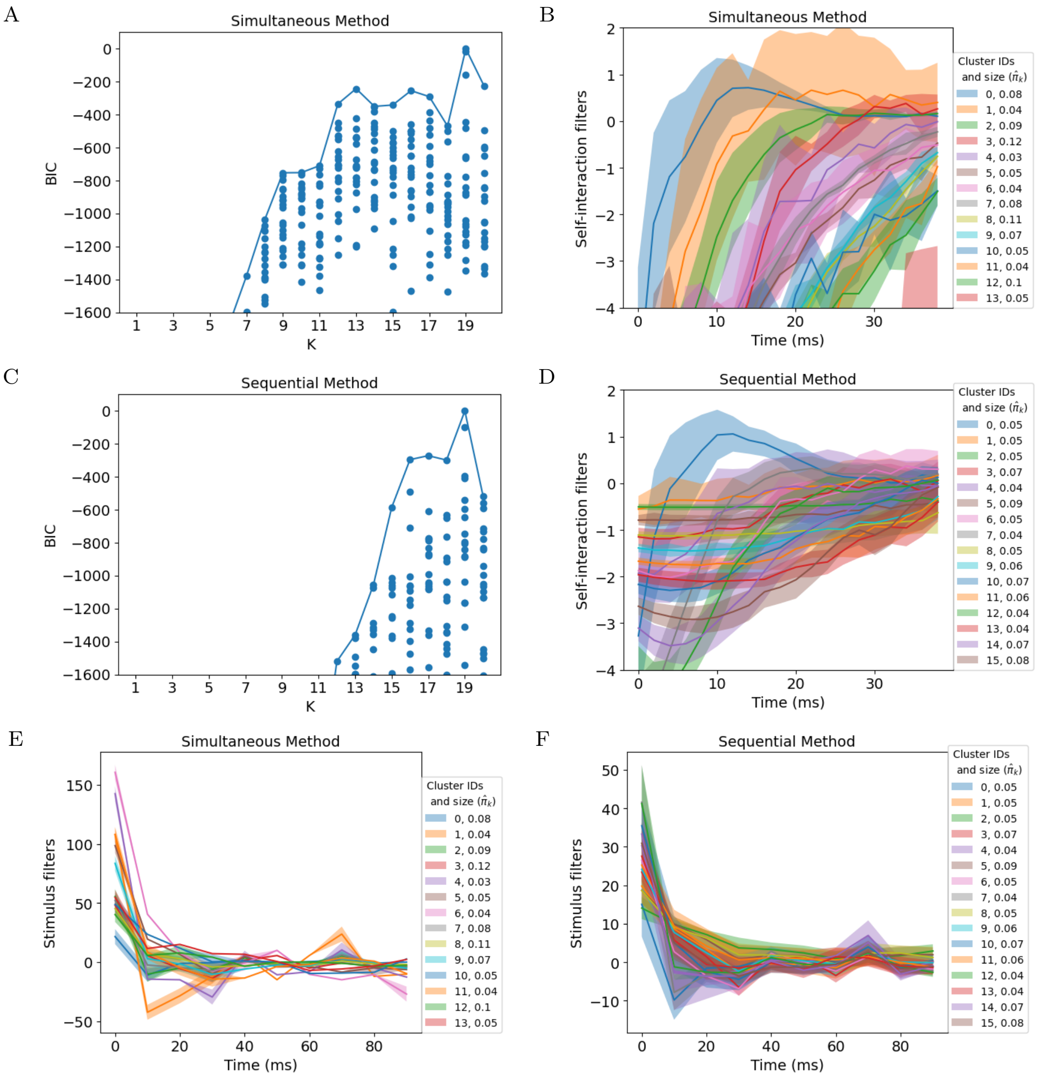

Fig C: **Allen Cell Types Database Dataset, Alternative Model.** A-D: Same analysis as in Fig 4, but with the alternative model (case B) where all parameters are cell-type dependent. Here, $\hat{K} = 19$ is selected by BIC for both methods. In addition, panels E and F show comparable information to B and D, but for the stimulus filters.

both simultaneous and sequential approaches and performed model selection on three of the four folds using the Noise 1 stimulus. We then fit GLM models for each neuron in the test fold using the Noise 1 stimulus with the results of training (selected hyperparameters, as well as the fitted hierarchical model for the simultaneous method). Then, we use the Noise 2 stimulus to evaluate those test neurons. We repeat this process for each choice of test fold to evaluate all neurons.

To generate panels B, D, and F:

- We fix all hyperparameters to their values selected when using all presentations of Noise 1 to all neurons.
- We split the neurons into four folds, and applied both simultaneous and sequential approaches to subsets of the folds: either one fold, or two folds, or three folds. These are the columns of panels B, D, and F. We also used only one, two, or three

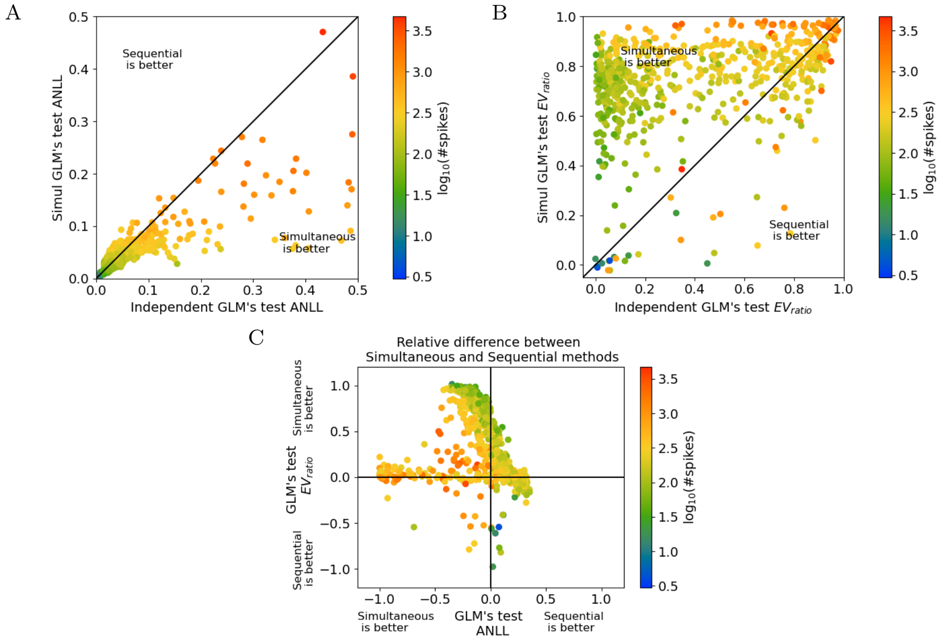

Fig D: **Allen Cell Types Database Generalization Performance, Alternative Model.** Same analysis as Fig 5A, 5C, and 5E, but with the alternative model (case B) where all parameters are cell-type dependent. Results here are comparable to those in the main manuscript.

presentations of the Noise 1 stimulus: these are the rows of panels B, D, and F. We then estimate the GLM parameters and cluster assignments for all neurons in the test fold(s), using all presentations of the Noise 1 stimulus with the fixed hyperparameters and the results of training (the fitted hierarchical model for the simultaneous method or the fitted GMM for the sequential method). We then evaluated ANLL and EV_{ratio} on all presentations of Noise 2 to these test neurons. This process is then repeated for each possible division of the folds into training and testing, and ANLL and EV_{ratio} for each neuron are each averaged over all divisions in which that neuron was in the test fold(s).
For instance, to obtain the top left square of panel B, we repeatedly applied both simultaneous and sequential approaches to 1/4 of the neurons (one fold) using only one presentation of the Noise 1 stimulus. Then, for the remaining 3/4 of the neurons (three folds), we estimated their GLM parameters using all presentations of Noise 1 (along with the fitted hierarchical model for the simultaneous method), and finally evaluated them using all presentations of the Noise 2 stimulus.

- In each cell of panels B and D, we define a *sample* as the relative difference (simultaneous minus sequential, divided by their sum) in ANLL and EV_{ratio}, respectively, of a single neuron.
- In each cell of panel F, we define a *sample* as the relative difference (simultaneous minus sequential, divided by their sum) in ARS of cluster assignments between a pair of different divisions of the neurons into training and testing.
- In each cell, the color is the median across samples, and we place an asterisk if the simultaneous method is significantly better (negative relative difference for panel B, positive for D and F), i.e. if a one-sided Wilcoxon signed-rank test of the samples produces an uncorrected p-value less than 0.001.

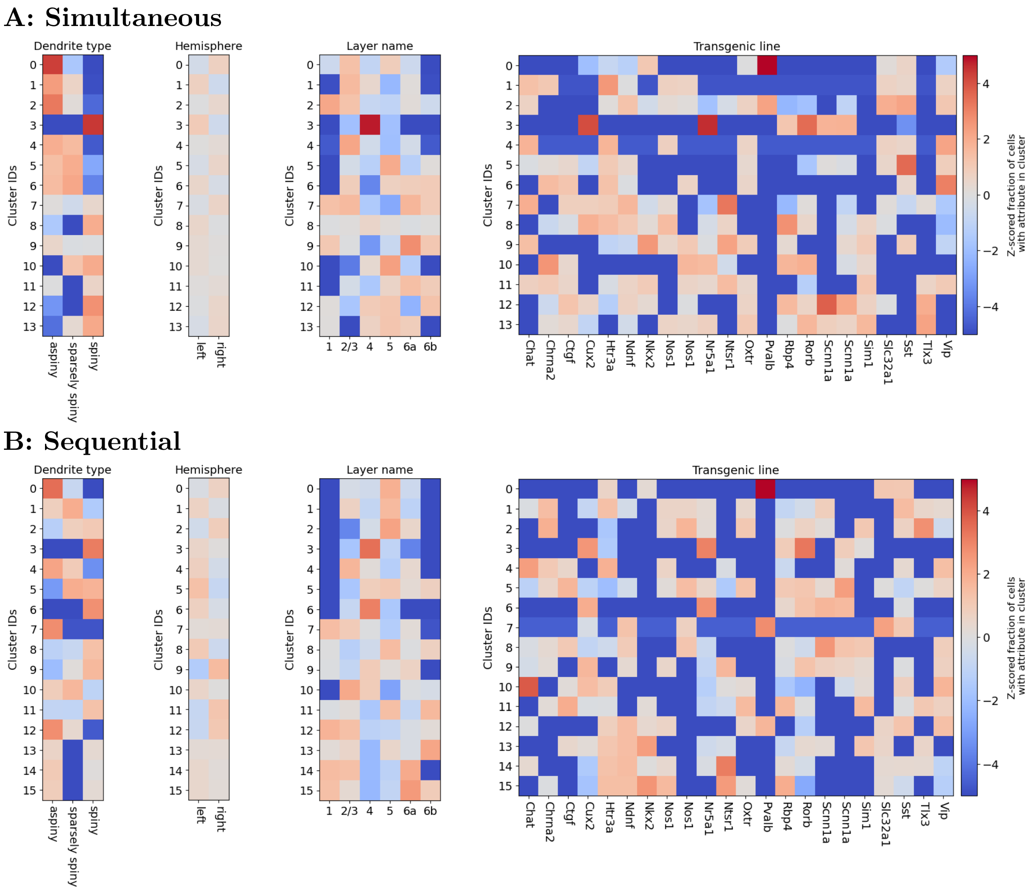

Fig E: **Allen Cell Types Database Metadata, alternate model.** Same analysis as Fig 6, but with the alternative model (case B) where all parameters are cell-type dependent. Results here are comparable to those in the main manuscript.

- Between each pair of adjacent cells, except horizontal pairs in panel F, we computed a two-sided Wilcoxon signed-rank test on the paired differences between their samples. We placed a dash if the p-value is less than 0.001.
- The p-values reported in the caption for trends in horizontal and vertical differences, except for the horizontal trend in panel F, are calculated by a one-sided Wilcoxon signed-rank test on the paired differences between samples, aggregated across all six pairs of either horizontally or vertically adjacent cells. P-values greater than 0.1 are reported as “not significant.”
- Between each pair of horizontally adjacent cells in panel F, samples cannot be paired, so we used a two-sided, two-sample t-test between the samples from the left and right cells to measure significance, and placed a dash if the p-value is less than 0.001.
- Likewise, the p-value for the horizontal trend in panel F is computed by a one-sided two-sample t-test between the aggregated samples from the six leftmost cells and the six rightmost cells (it was greater than 0.1 and thus labeled “not significant”).

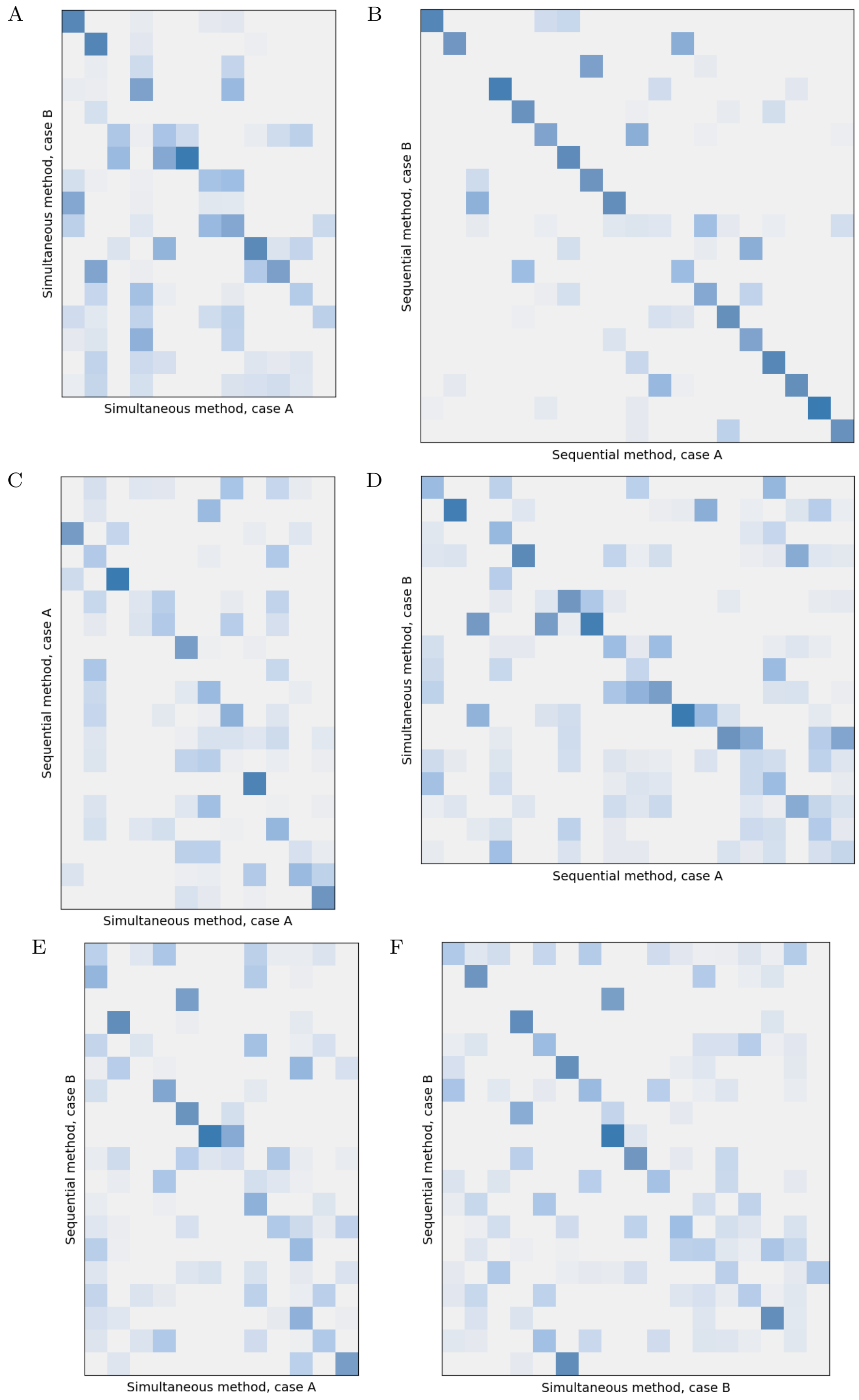

Fig F: **Similarity of clusterings from the four method-case combinations.** Each panel shows the confusion matrix of the clusterings from two of the four method-case combinations.

D Further Details for Simulated Data

This section details our process for simulating datasets from hierarchical models in both cases A and B. These datasets were used to generate Figs 2 and 3 and Figs A and

B respectively.

Instead of truly sampling the cell-types, k_i, from $\{\pi_k\}$ we simply assign an equal number (40) of neurons to each class. To set the filters, we use functions that parameterize the stimulus filters as a decaying exponential,

$$g(t; a_{stim}, \tau_{stim}) \equiv a_{stim} e^{-\frac{t}{\tau_{stim}}}, \tag{M}$$

and the self-interaction filters as the sum of a negative decaying exponential and a Gaussian bump,

$$h(t; t_{ref}, \tau_{ref}, a_{ISI}, \mu_{ISI}, \sigma_{ISI}) \equiv -e^{-\frac{t-t_{ref}}{\tau_{ref}}} + a_{ISI} e^{-\frac{(t-\mu_{ISI})^2}{2*\sigma^2_{ISI}}}. \tag{N}$$

In both cases A and B, $\boldsymbol{\mu}_k^{\text{self}}$ are determined with $t_{ref}, \tau_{ref}, a_{ISI}, \mu_{ISI}, \sigma_{ISI}$ that are set to uniformly spaced values for each k. In case A, a_{stim}, τ_{stim}, and β_k^0 are each fixed to a single value for all neurons, whereas in case B they are also set to uniformly spaced values for each $\boldsymbol{\mu}_k$. All relevant constants that parameterize this spacing are enumerated in Table A.

Evenly spaced params for $\boldsymbol{\mu}_k^{\text{self}}$ in both cases A and B			
Parameter	Value for $\boldsymbol{\mu}_1$	Increment	
t_{ref}	2	1.75	
τ_{ref}	2	0.5	
a_{ISI}	0.2	-0.05	
μ_{ISI}	3	2	
σ_{ISI}	3	0.25	
Evenly spaced for $\boldsymbol{\mu}_k$ in case B, fixed to one value for all $\boldsymbol{\beta}_i$ in case A			
Parameter	Value for $\boldsymbol{\mu}_1$ (case B)	Increment (case B)	Value for all $\boldsymbol{\beta}_i$ (case A)
a_{stim}	0.5	0.125	0.9
τ_{stim}	4	0	4
μ_k^0	-4.5	0.25	-5

Table A: Spacing of the parameters used to simulate datasets for cases A and B.

We also used the same stimulus downsampling factor, $d^{\text{stim}} = 5$, in our simulations. In order that its value does not affect the simulation much, we compute $g(t), t \in \{1, .., T^{\text{stim}} * d^{\text{stim}}\}$, and then sum the τth block of d^{stim} values, $\sum_{t=(\tau-1)*d^{\text{stim}}}^{\tau*d^{\text{stim}}} g(t)$ to assign to $\beta_i^{\text{stim}}(\tau)$ (case A) or $\mu_i^{\text{stim}}(\tau)$ (case B).

Once all $\boldsymbol{\mu}_k$ have been set, $\boldsymbol{\beta}_i$ are sampled from $f(\boldsymbol{\beta}_i; \boldsymbol{\mu}_k, \sigma^2 * I)$, where they have not already been determined (case A). Once all of $\boldsymbol{\beta}_i$ has been determined, the spike train for the ith neuron is then simulated by sampling from the GLM's distribution for $y_i(1)$, then $y_i(2)$, all the way up to $y_i(T_i)$ (using eq 2).

When $\sigma > 10^{-5/6}$ is used, the simulation becomes unstable, because some $\boldsymbol{\beta}_i^{\text{self}}(\tau)$ values become high enough to cause runaway feedback, whereby the number of spikes in bins spaced τ apart diverge to infinity with increasing time. This critical value of $\boldsymbol{\beta}_i^{\text{self}}(\tau)$ is around $-[\beta_i^0 + \max_t x_i(t) \sum_{t=1}^{T^{\text{stim}}} \beta_i^{\text{stim}}(t)]$, such that a single spike τ time bins ago can raise the spike rate above 1 if it occurs during an extended period of high stimulus values. To prevent this scenario, we impose $y(t) \in \{0, 1\}$ while simulating (as in [2]), and do not consider such high values of σ that would lead to such severe model misspecification that the true model parameters cannot be recovered. It is worth noting that this problem can arise even for filters fitted to real neural data because of model misspecification - specifically, there is a maximum number of times any neuron can spike in a given time bin because of its absolute refractory period, whereas the (Poisson) GLM makes no such assumption.

D.1 Simulating spike trains from fitted models

Here, we consider the spike trains generated from GLM models whose parameters are taken from fits to the Allen Cell Types Database, and models fitted to those spike trains. This analysis provides an extension of the simulation studies in Sections 3.1 and B.2, asking how accurate the simultaneous method is in the parameter regime appropriate for the Allen Cell Types Database.

As discussed in Section D, certain GLM parameter regimes will lead to severe model misspecification because of the truncation of Poisson-generated spike counts to at most 1 spike per bin. Therefore we first select the five clusters from those fitted and displayed in Fig CB and CF that we expect to produce a sufficient number of spikes, while requiring the fewest instances where the truncation of the Poisson distribution affects the simulated data. To do this, we compute the maximum spiking rate that could arise from simulating a GLM with the kth cluster mean as parameters, following an isolated spike:

$$\log(\text{rate}_{\max}) = \max_{\tau} \hat{\mu}_k^{\text{self}}(\tau) + \hat{\mu}_k^0 + \max_t x(t) \sum_{t=1}^{T^{\text{stim}}} \hat{\mu}_k^{\text{stim}}(t).$$

As discussed in Section 3.1, when this quantity is above 0 the spike rate τ time bins after a spike will be greater than 1, leading to model misspecification. We thus choose the five clusters with the smallest $\log(\text{rate}_{\max})$, provided it is two standard deviations (determined using the fitted $\hat{\Sigma}_k$) above -2. The results of fitting the sequential and simultaneous methods to the data simulated from these clusters is shown in Fig G, and generally shows that, while imperfect, the simultaneous method outperforms the sequential method. It also appears that the major mistake the simultaneous method makes in this instance, merging two clusters with very similar self-interaction filters, is a reasonable one.

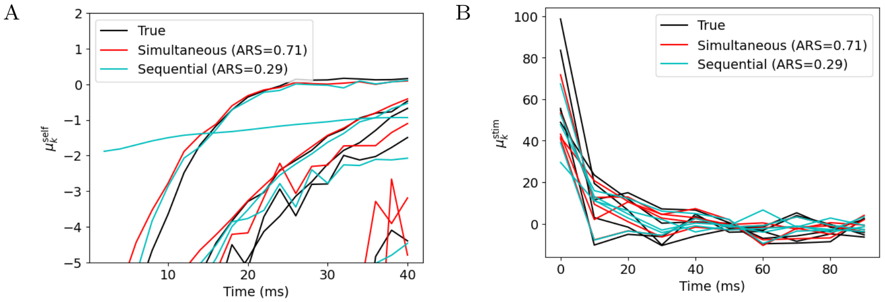

Fig G: **Simulated data from cluster models with fitted parameters.** The simultaneous method adequately recovers the true cluster structure of simulated data. Here, the true cluster structure is that fitted using the simultaneous method.

E Model Selection

E.1 Alternative Model Selection: Validation Log-Likelihood

In addition to using BIC to perform model selection, we also investigate using the validation log-likelihood (VLL) on held out neurons (see section 2.4 detailed method).

For our simulated data, we use the exact same models that the BIC analysis was applied to (Figs 3 and B), but evaluate the model log-likelihood, averaged over a new validation set of 10 neurons per true cluster. Unlike with BIC, this VLL increases

monotonically with K towards an asymptote for both the simultaneous and sequential methods.

For each of the 50 simulated datasets, we selected the lowest K whose VLL was within one standard error of the maximum, where this standard error was measured over the 50 datasets, after subtracting off each dataset's VLL for $K = 1$. This last step improves performance because each dataset has an offset in VLL that results from the spike density in the validation neurons (compare Figs I and J for an illustration of this effect in the Allen Cell Types Database data). Overall, this approach can be thought of as a variant of the one-standard-error (1SE) rule [3]. These results are shown in Fig H, and can be compared to those obtained via BIC in Figs 3 and B. For case A, BIC is better, but for case B, the results are more mixed. Across all cases, BIC tends to select a lower $\hat{K}$ than VLL.

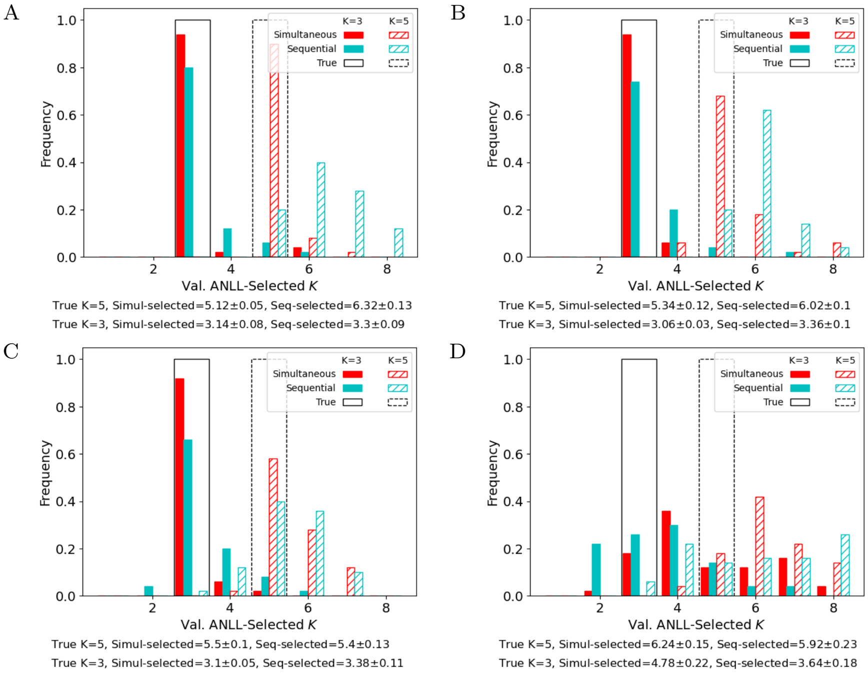

Fig H: **Model selection of $\hat{K}$ using validation loss.** Frequencies of $\hat{K}$ estimated via the loss on held out neurons over 50 simulated datasets with the same μ_k as in Fig 2 (case A, panels A and B) or Fig A (case B, panels C and D), and $\sigma = 10^{-2}$ (A and C) or $10^{-5/6}$ (B and D), the maximum value that does not result in degenerate simulations. Black lines indicate true K. Summary below plots gives the mean ± SEM of estimated $\hat{K}$ across the 50 datasets for each case and each method.

For real data, where the true distribution of neurons is unknown, we must use cross-validated log-likelihood (CVLL) to get a metric that is not biased by our choice of test neurons. To compute CVLL, we randomly partition the neurons into L equally-sized sets $A_1, ..., A_L$. Each set A_l in turn is held out while the parameters are fit using K clusters to the remaining neurons, and then the validation loss is simply $\frac{1}{|A_l|} \sum_{i \in A_l} \mathrm{LL}_i$ (see Section 2.4 for our definition of LL_i). CVLL is then evaluated as the average validation loss over the L different sets.

When this approach is applied to the Allen Cell Types Database data, we observe the same issue with monotonic improvement in VLL with K (Fig I). Additionally, the simultaneous method yields VLLs for each validation fold that are offset far away from

one another (Fig IA and IB). These offsets stem from large differences between the spike trains of individual neurons: some (with many spikes) yield a much higher ANLL, while others (with few spikes) yield a low ANLL (see Figs 5 and D for an illustration of this phenomenon). Since the ANLL of all the validation cells factors into the validation loglikelihood (VLL) in the simultaneous method, a random partition of the neurons into different validation sets produces some sets with higher VLL and some with lower VLL.

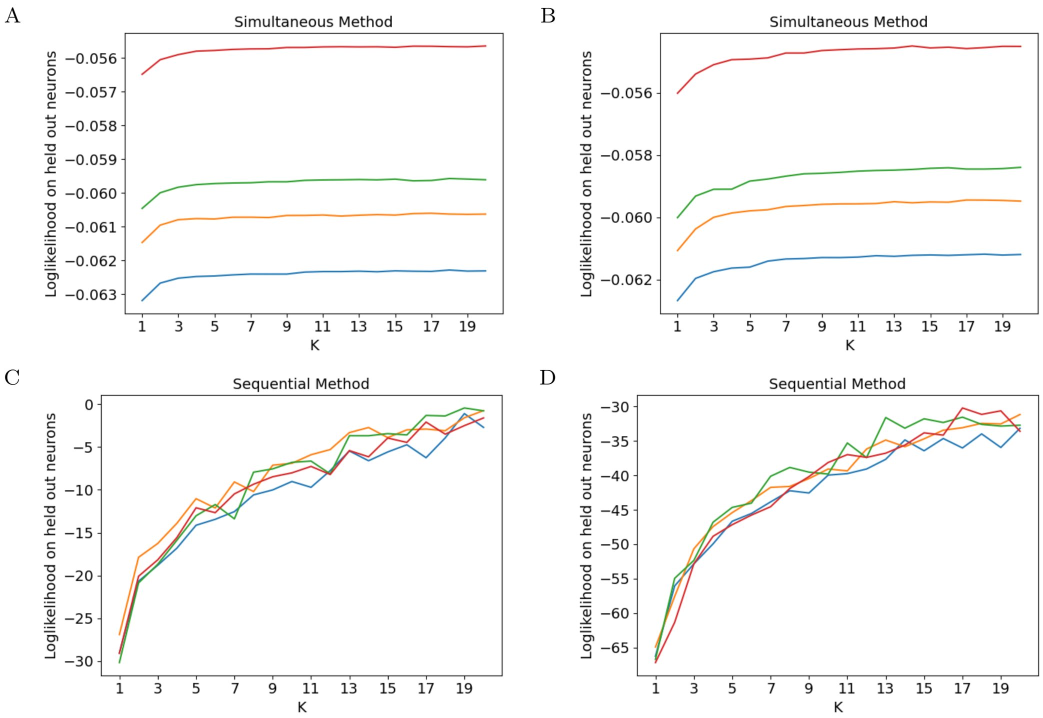

Fig I: **Validation Log-Likelihood on Allen Cell Types Database Dataset.** Each color represents a specific split of the neurons into training and testing. All log-likelihoods increase monotonically with K. The different folds of the simultaneous method are heavily offset relative to one another as a result of the variety of spike counts in each fold's validation set (see Figs 5 and D).
A: Case A, Simultaneous
B: Case B, Simultaneous
C: Case A, Sequential
D: Case B, Sequential

To account for the offsets between these curves, we first shift them by subtracting off their values at $K = 1$ before using the standard 1SE rule to select $\hat{K}$ (Fig J). That is, we pick the lowest K whose CVLL is within one standard error of the maximum, where the standard error is computed over the shifted VLLs of the different partitions of the neurons into training and validation.

Just as with the simulated datasets, CVLL selects a much higher $\hat{K}$ than BIC for each case (compare to Fig 4A and 4C and CA and CC). It is worth noting that as the VLL curves have not fully plateaued, repeating this analysis with a greater range of K would likely result in a higher selected $\hat{K}$.

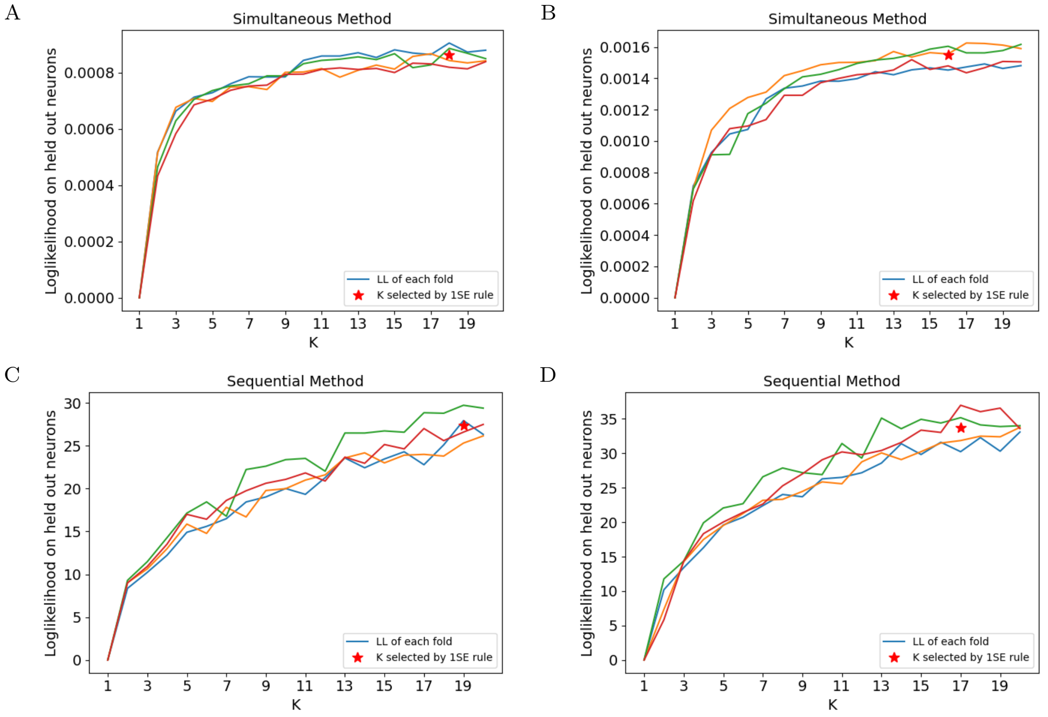

Fig J: **Validation Log-Likelihood on Allen Cell Types Database Dataset.** Same colors as Fig I. The One-Standard-Error rule (1SE) applied to the Cross-Validated Loss (CVL) selects very high $\hat{K}$ for all methods (red stars). To account for the large offsets shown in Fig I, each VL curve is first shifted (as shown here) so that its value for $K = 1$ is 0 before 1SE is applied.
A: Case A, Simultaneous
B: Case B, Simultaneous
C: Case A, Sequential
D: Case B, Sequential

E.2 Generalization Performance of Model Selection Metrics

We have now shown two different approaches to model selection and how they differ, both in method and results on simulated and real data. For the simulated data, as we know the true K, we are able to assess how the accuracy of selected $\hat{K}$. For the Allen Cell Types Database data, we cannot do this directly as there is no ground truth for K, but we can attempt a similar approach to that used in Figs 5 and D, using the same measures of performance on predicting responses to the test stimulus of neurons that were held out from fitting the cluster models. Just as we argued that improvements in these metrics implied improved estimation of model parameters, $\boldsymbol{\beta}_i$, we can look for differences in these metrics, evaluated on test neurons, with respect to the K used to fit the simultaneous method, and compare them to model selection criteria computed on the train/validation neurons. The $\boldsymbol{\beta}_i$ estimated by the sequential approach do not depend on K, so this analysis is limited to the sequential method.

However, these metrics do not show sufficient dependence on K to warrant such an analysis (Fig K). With the possible exceptions of $K \in \{1, 2\}$, there is very little difference between the two cases, A and B, or between fitted K, relative to the variation between neurons.

F Cluster Distribution for Sequential Method with BIC-selected K

In Fig 4, we showed the cluster distributions for $K = 12$ for both sequential and simultaneous methods to facilitate comparison between the two. However, $K = 19$ is the BIC-selected value for the sequential method, so, for completeness, we show those clusters in Fig L. The results are much like those for $K = 12$, with highly overlapping clusters bunched near 0.

References

1. Murphy KP. Machine learning: a probabilistic perspective. Adaptive computation and machine learning series. Cambridge, MA: MIT Press; 2012.

2. Weber AI, Pillow JW. Capturing the Dynamical Repertoire of Single Neurons with Generalized Linear Models. Neural Computation. 2017;29(12):3260–3289. doi:10.1162/neco_a_01021.

3. Hastie T, Friedman J, Tibshirani R. The Elements of Statistical Learning. Springer Series in Statistics. New York, NY: Springer; 2001. Available from: http://link.springer.com/10.1007/978-0-387-21606-5.

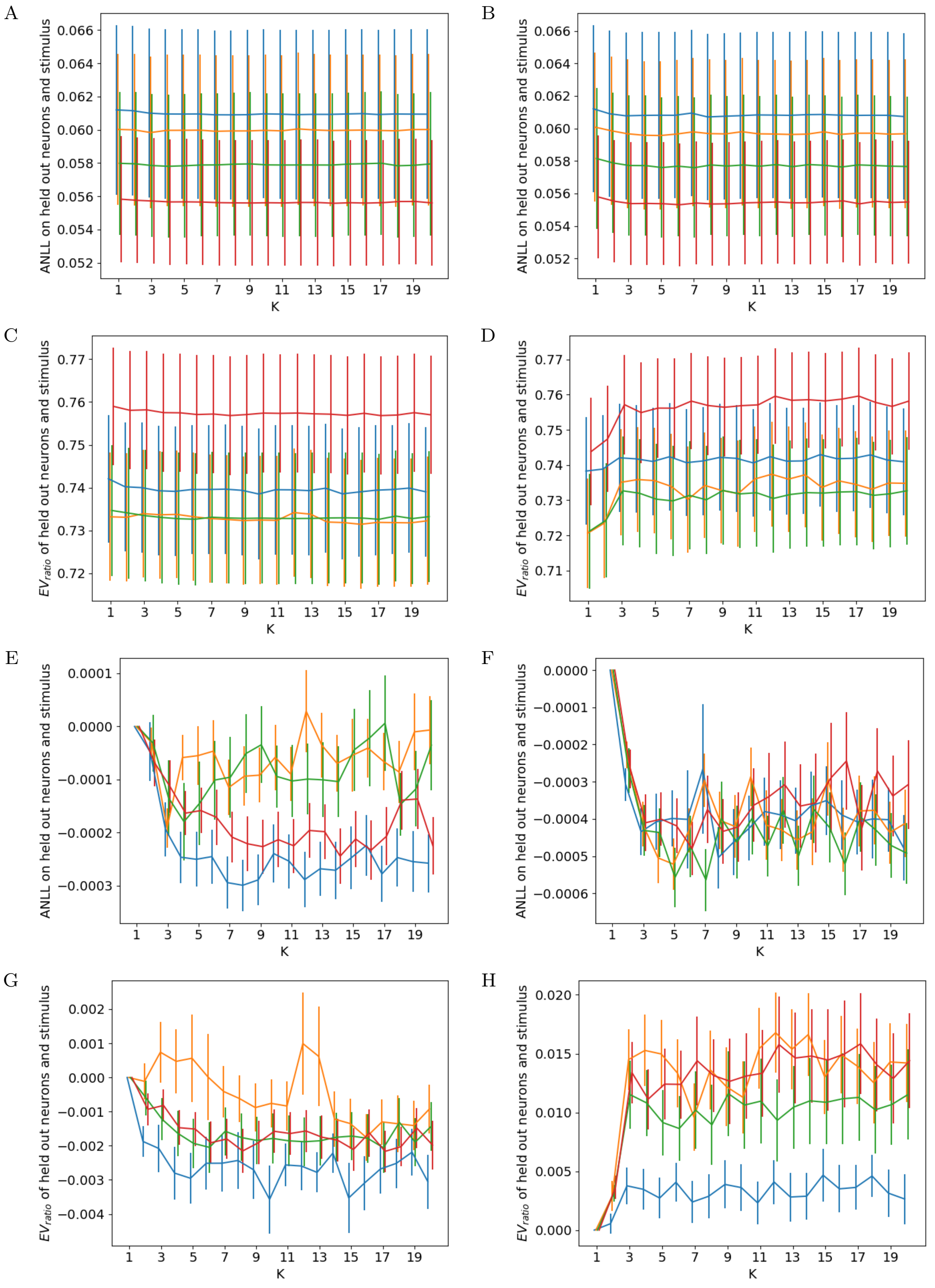

Fig K: **Model Comparison using CVL on Allen Cell Types Database Dataset.** Validation metrics for each fold are shown, with the same colors as in Fig I. A-D: Error bars are one SE across neurons in the validation fold. E-H: The curves are shifted so that their values at $K = 1$ are 0, to facilitate judging their (lack of) change w.r.t. K. Error bars are one SE across validation neurons of the shifted metrics.
A,E: Case A, ANLL
B,F: Case B, ANLL
C,G: Case A, EV_{ratio}
D,H: Case B, EV_{ratio}

A

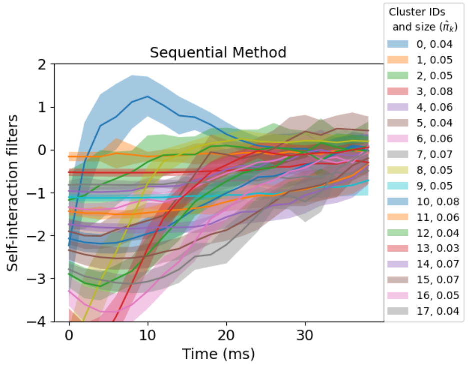

Fig L: **Clusters discovered by the sequential method with $K = 19$ are highly overlapping and tightly bunched near 0.**
