# Supplementary material for: Modeling functional cell types in spike train data
Source: PLoS Comput Biol. 2023 Oct 12;19(10):e1011509. doi: 10.1371/journal.pcbi.1011509 (PMC10569560; doi:10.1371/journal.pcbi.1011509)
Supplement: Attachment — Submitted filename: review_Zdeblick.pdf [file pcbi.1011509.s002.pdf]

Review of "Modeling functional cell types in spike train data"

Summary

The authors define a hierarchical generative model for cell types, single-cell parameters, and neural responses, to simultaneously estimate cell types and fit single-cell models. Earlier proposals sequentially fitted single-cell models and then clustered the obtained parameter point estimates into cell types. Here, the authors do it the 'proper Bayesian way' and marginalize over the latent variables of the single-cell models. The hierarchical Bayesian approach is not only statistically more sound, but also increases performance of predicting single-cell activity. The paper is well written and I enjoyed reading it.

Main concern

My only major issue is the model selection and how you end up with 'simultaneous case A' as the preferred model. Based on the best BIC values in Figs 4A,C and S3A,C the four models ('simultaneous case A', 'sequential case A', 'simultaneous case B', 'sequential case B') perform equally well (for their respective optimal K). Maybe one shouldn't just look at BIC, and in Figs 5 and S4 you show that the simultaneous model outperforms the sequential one in both cases. However, why is 'simultaneous case A' preferable over 'simultaneous case B'? Is it just that Fig 4B looks nicer than Fig S3B? While you state in section S2.3 that "the results are comparable to those in the main manuscript", this is not at all the case for these 2 panels.

Why is $\sqrt{\Sigma_{self}}$ in Fig S3B much larger than in 4B, whereas it's similar for S3D and 4D?

Please add the stimulus filters to Fig S3! Are these indeed virtually identical, as your choice for the simulations (Fig 2A) seems to suggest? Otherwise I could imagine a situation as sketched in my Figure 1, where I reduced the dimension of β_{self} and β_{stim} to 1 each. Clustering β_{self} ignores

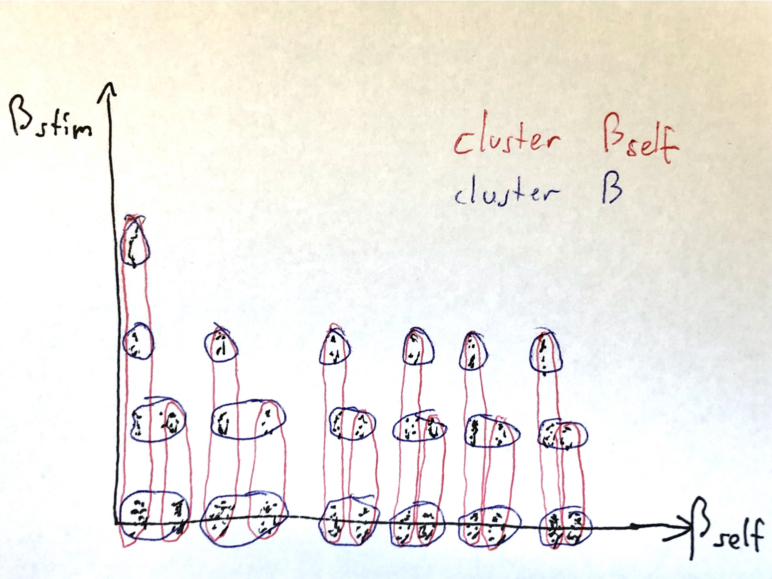

Figure 1: A sketched hypothetical case where clustering the data points using β_{self} yields 12 clusters with small $\sqrt{\Sigma_{self}}$ and clustering β yields 19 clusters with bigger $\sqrt{\Sigma_{self}}$.

(the variance in) β_{stim} and yields clusters with small $\sqrt{\Sigma_{self}}$. Clustering the full β yields different cluster assignments with bigger $\sqrt{\Sigma_{self}}$ (but smaller $\sqrt{\Sigma_{stim}}$), in particular for those clusters with large number of neurons.

It would be worthwhile to add a supplementary figure showing how well the cluster assignments of the 4 models agree. Plot the binary $N \times K$ matrices of the cluster assignments (or real soft assignments if you wish) using the same ordering of neurons obtained from the 'simultaneous case A' model.
Fig 6 and S5 try to do that to some extend, but it is hard to judge the similarity given the ordering. Cluster #0 is apparently the same for all four panels. However, cluster #1 in 6A corresponds to #4 in 6B, #2 in S5A, and #7 in S5B...

Minor comments

page 2, Introduction: I was bit tripped up by the first sentence. In the spirit of George Box, while the model will be wrong, it should not only be tractable **but useful**. I'd add something like *...allow for tractable modeling, while still capturing relevant aspects of ...*

page 4, section 2: You did not "develop" a Bayesian information criterion, you use the standard one (e.g. wikipedia) but for unclear reasons multiply it by $-\frac{1}{2}$.

page 5: Why is it ok to use your cross-validation scheme (i.e. there is no concern of data leakage) in spite of dealing with time-series data?

page 6, Eq (5): The constraint $\sum_k \pi_k = 1$ is missing.

page 6, Algorithm 1: Wrong signs in front of the ℓ_2-regularization terms. Has to be $-$ not $+$, as in Eq (3), cause you are maximizing not minimizing.

page 6, Algorithm 1: How do you initialize $\hat{\Omega}_k$ for the non-convex GMM problem?

page 7, $\sim$middle: As already stated, please plot β_{stim} as additional panels in Fig S3 to support that you can indeed "assume that β_i^{stim} and β_i^0 are independent of the cell type k".

page 7, before Eq (11): Strictly speaking, spiking activity of each neuron is independent *conditioned on the stimulus.*

page 8, Fig 1B: Wrong symbols for spiking response (x should be y) and stimulus (s should be x).

page 8, E-step: What was the reason to use the Laplace approximation with diagonal covariances? Wouldn't mean field variational inference, as e.g. Blei et al. do for the GMM in "Variational Inference: A review for Statisticians", yield a better approximation?

page 10, Alg 2: Shouldn't the update for $c_{i,k}^{-1}$ be $\text{diag}\left((\nabla^2 \log P)^{-1}\right)^{-1}$, as in the supplementary material? Diagonalize the covariance, not the precision matrix?

page 10, Algorithm 2: How do you initialize $\hat{\Omega}_k$ for the non-convex GMM problem?

page 12, section 3.1: typo, $10^-5/6$ should be $10^{-5/6}$.

page 13, Fig 2F: Is there an intuitive explanation why $\sqrt{\Sigma}$ saturates for small σ?

page 14, Fig 3: Is there an intuitive explanation why simultaneous model underestimates whereas sequential model overestimates K?

page 14, right before section 3.2.1: (weights 0.6-0.12) should be (0.06-0.13) if the legend in Fig 4 is correct.

page 15, Fig 4: According to panel C, the correct K for the sequential method is $K = 19$. How would panel D look without forced merging of clusters, i.e. for $K = 19$?

page 16, section 3.2.2, 2nd paragraph: "Many metadata labels belonged to certain clusters discovered by the *sequential* method..." ⇒ "Many metadata labels belonged to certain clusters discovered by the *simultaneous* method..."

Some references are now available not only as preprints but as (hopefully improved) peer-reviewed articles.

page 21: ref 1 has been published in Nature Neuroscience, ref 13 in Neural Computation.

page 22: ref 18 has been published in NeurIPS (I know, not really a journal), ref 19 in Cell, and ref 26 in Nature Neuroscience.

GitHub: I applaud you for making the code to reproduce the analysis available. However, some scripts won't run through, e.g. N in data_grabber.py is not defined yielding a NameError.
Please add a license to the repo, otherwise (see https://choosealicense.com/no-permission/) only you as creator are permitted to use the software.
