# Supplementary material for: Modeling functional cell types in spike train data
Source: PLoS Comput Biol. 2023 Oct 12;19(10):e1011509. doi: 10.1371/journal.pcbi.1011509 (PMC10569560; doi:10.1371/journal.pcbi.1011509)
Supplement: Attachment — Submitted filename: Plos CB Response to Reviewers.pdf [file pcbi.1011509.s003.pdf]

Reviewer #1: The authors define a hierarchical generative model for cell types, single-cell parameters, and neural responses, to simultaneously estimate cell types and fit single-cell models. Earlier proposals sequentially fitted single-cell models and then clustered the obtained parameter point estimates into cell types. Here, the authors do it the 'proper Bayesian way' and marginalize over the latent variables of the single-cell models. The hierarchical Bayesian approach is not only statistically more sound, but also increases performance of predicting single-cell activity. The paper is well written and I enjoyed reading it.

Response: We thank the referee for this extremely thorough review. We feel the paper is much stronger after addressing these points and we appreciate the referee's care and attention.

Main concern

1) My only major issue is the model selection and how you end up with 'simultaneous case A' as the preferred model. Based on the best BIC values in Figs 4A,C and S3A,C the four models ('simultaneous case A', 'sequential case A', 'simultaneous case B', 'sequential case B') perform equally well (for their respective optimal K). Maybe one shouldn't just look at BIC, and in Figs 5 and S4 you show that the simultaneous model outperforms the sequential one in both cases. However, why is 'simultaneous case A' preferable over 'simultaneous case B'? Is it just that Fig 4B looks nicer than Fig S3B? While you state in section S2.3 that "the results are comparable to those in the main manuscript", this is not at all the case for these 2 panels.

   Response: This is an important issue touching on model selection, use, and interpretation, and we appreciate the opportunity to improve our manuscript by revising it accordingly. We have added a clarifying paragraph at the end of Section 2.4:

   "We note here that these model selection criteria assume a specific form of model, and thus do not admit direct comparisons between the simultaneous and sequential methods, or with the alternative model we consider in Section \ref{sec:case-B}. All plots displaying model criteria are normalized so that the optimal value is at 0 to facilitate judging the scale of the plot. All direct comparisons between methods must therefore rely on the evaluation metrics we consider in the following Section."

   Accordingly, we use our evaluation metrics to argue that the simultaneous method is better than the sequential method. We could also use them to compare case A vs case B, but the results are not as striking and this is not the main focus of our work. We elaborate on this, as well as our choice to put case A in main and B in the supplement in new paragraphs at the beginning of Section S2:

   "We note here that providing analysis of the relative benefits of case A and case B is not a major objective of this work. Rather, we seek to demonstrate only that there are options for how one chooses to define a hierarchical model within our general framework, and each may have its own benefits and drawbacks, depending on the line

of inquiry. As the rest of this section will elaborate, this option is equally present for both the simultaneous and sequential methods.

“We focus on case A in the main primarily because it provides a simpler model than B: it selects fewer clusters, lower within-cluster variances (Σ_k), and by its definition is a lower dimensional model. All of these factors give case A an advantage over case B in terms of interpretability. Additionally, case A performs better than B in terms of model selection on simulated data (compare Figures \ref{fig:repsim_modsel2} and \ref{fig:repsim_modsel_VL}A,B with Figures \ref{fig:repsim_modsel_all} and \ref{fig:repsim_modsel_VL}C,D). Finally, the shared variables in case A, the self-interaction filters, are more strongly associated with intrinsic neural dynamics, whereas, at least in the IVSCC dataset, the stimulus-filters may depend more on other factors, such as the impedance of the electrode-neuron interface used to administer the stimulus. This, along with the fact that case A is lower dimensional, may account for any relatively better performance by case A. However we also acknowledge that there are cases where case B may be preferable, such as when one specifically wishes to distinguish the stimulus processing properties of neurons.”

2) Why is $\sqrt{\Sigma}$_self in Fig S3B much larger than in 4B, whereas it’s similar for S3D and 4D?

   Response: The next three comments (including this one) are beautifully posed, and provide an excellent scaffold for answering this question, as well as addressing the results shown in the requested figures. This explanation now makes up most of section S2.3 - we have broken it down and simplified it to address these comments here.

   In short, the addition of stimulus filters changes cluster assignments much more for the simultaneous method than for the sequential method, as seen in the new Figure S6, which more directly addresses your later comment. Because the clustering assignments change, the position and spread of the clusters change. Generally the spread increases because, when only self-interaction filters are clustered, the cluster assignments are optimized to allow for minimum cluster spread along those dimensions, since that will minimize the loss of the hierarchical model.

   As for why the clustering assignments change for the simultaneous, but not sequential method, we address this in response to the next comment.

3) Please add the stimulus filters to Fig S3! Are these indeed virtually identical, as your choice for the simulations (Fig 2A) seems to suggest? Otherwise I could imagine a situation as sketched in my Figure 1, where I reduced the dimension of βself and βstim to 1 each. Clustering βself ignores (the variance in) βstim and yields clusters with small $\sqrt{\Sigma}$self . Clustering the full β yields different cluster assignments with bigger $\sqrt{\Sigma}$self (but smaller $\sqrt{\Sigma}$stim), in particular for those clusters with large number of neurons.

   Response: These have been added as Figure S3 E and F. Panel F (sequential) seems to show that they are indeed virtually identical, resulting in relatively unchanged cluster memberships and similar cluster positions in the self-interaction filter dimensions. Panel

E, however, shows that the simultaneous method finds much more distinction between stimulus filters, leading to the greater change in clustering assignments.

4) It would be worthwhile to add a supplementary figure showing how well the cluster assignments of the 4 models agree. Plot the binary N × K matrices of the cluster assignments (or real soft assignments if you wish) using the same ordering of neurons obtained from the 'simultaneous case A' model. Fig 6 and S5 try to do that to some extend, but it is hard to judge the similarity given the ordering. Cluster #0 is apparently the same for all four panels. However, cluster #1 in 6A corresponds to #4 in 6B, #2 in S5A, and #7 in S5B…

   Response: We agree that this information is useful, and added confusion matrices comparing the assignments of the 4 models in Section S2.3 and the new Figure S6. We believe this conveys the information more appropriately, since N is large and therefore N x K matrices would be difficult to visualize. We also ordered the clusters to maximize the sum of the confusion matrix along the diagonal, which facilitates judging similarity, albeit making it more difficult to compare to Figures 6 and S5.

Minor comments

5) page 2, Introduction: I was bit tripped up by the first sentence. In the spirit of George Box, while the model will be wrong, it should not only be tractable but useful. I'd add something like ...allow for tractable modeling, while still capturing relevant aspects of …

   Response: Thank you for this important point - we have changed this sentence to include that the fitted models should also be useful:

   "A major goal of computational neuroscience is to build accurate models of the activity of neurons that can be used to interpret their function in circuits."

6) page 4, section 2: You did not "develop" a Bayesian information criterion, you use the standard one (e.g. wikipedia) but for unclear reasons multiply it by -½.

   Response: Thank you for this point - we've changed our language regarding this, now stating that we "use the Bayesian information criterion (BIC) to select hyperparameters". We use the formulation of BIC derived in Murphy, equations 5.30 or 8.57, which, as the referee notes, has a factor of -1/2 relative to the expression given on Wikipedia (as of the time of writing). Murphy refers to that formulation as the "BIC cost". Murphy's version comes from focusing on BIC as an approximation to the model evidence.

7) page 5: Why is it ok to use your cross-validation scheme (i.e. there is no concern of data leakage) in spite of dealing with time-series data?

Response: This is an important consideration, which we address by looking at the autocorrelations of spiking traces, with their smoothed trial average subtracted. The idea behind this is that if there is leakage across time, then fluctuations in the spiking response from the smoothed trial average will be correlated. When we smooth the trial average with a rectangular filter of 20ms or more, we get a fairly consistent picture of the autocorrelation of the residuals:

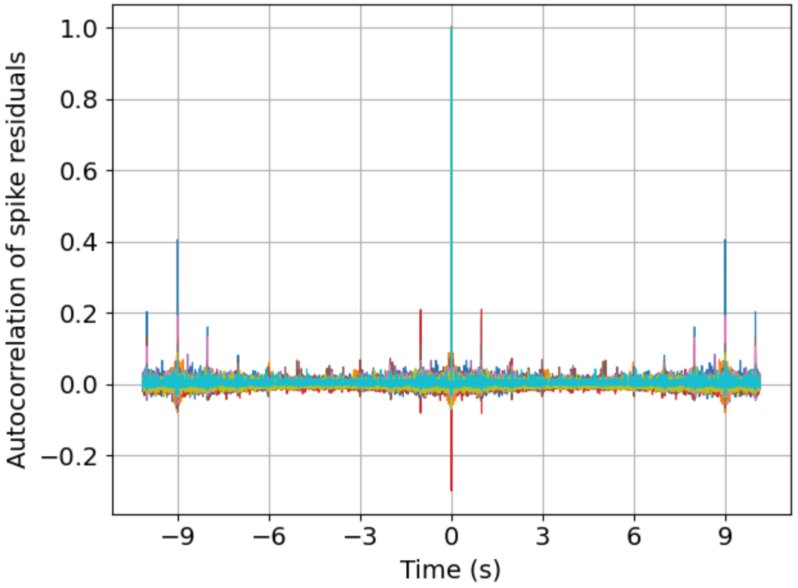

Indeed the correlations across time diminish very quickly. The spikes in the above plot at 1s and 9s correspond to structure in the presented stimulus: a given, 1 second long waveform is presented three times at three different amplitudes, making up what we refer to as a 9 second trial. Because we perform our cross-validation using these 9s trials, we also look at the correlation coefficients of these same residuals between pairs of trials, in order to more directly evaluate leakage at the trial-to-trial resolution. This results in the following histogram across neurons:

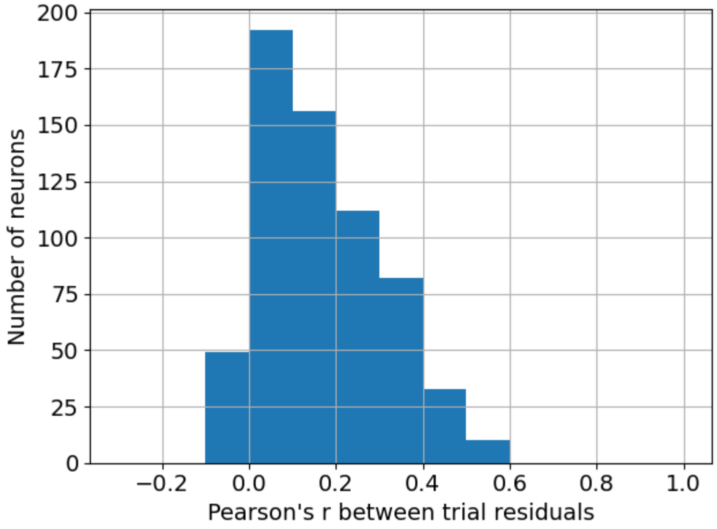

This plot shows that, while there is some small correlation between trials, as measured this way, there remains significant variability in trial responses. Some neurons do have moderate correlation values above 0.4, and in principle this could unfairly disadvantage the sequential method, which uses this cross-validation scheme to select hyperparameters. However, all of our experiments with simulated data use an oracle approach (instead of cross-validation) to select λ hyperparameters, and show clear advantages of the simultaneous method. It is also worth noting that ACTD results at the resolution of single neurons, such as in Figure 5 A, C, and E, show that the simultaneous method yields improvement for the overwhelming majority of neurons, not just a smaller fraction that might have higher correlation values in the plot above.

To address this concern in the paper, we added a couple sentences in our description of the sequential method's application to ACTD data in section 3.2 that better clarify what we did and communicate the assurances we make here:

"The GLM models are fitted with an ℓ_2 regularization, with hyperparameters selected using cross-validation, where individual presentations of the stimulus are used to define the partition of time bins (see \eqref{eq:lam-sel}). Autocorrelations of the residuals (after subtracting the smoothed trial average) indicated very low temporal correlations and the trial correlations of residuals were low for the majority of cells. "

8) page 6, Eq (5): The constraint sum_k π_k = 1 is missing.

Response: Thank you for this comment - we have added it to the paragraph preceding Eq (5)

9) page 6, Algorithm 1: Wrong signs in front of the `2-regularization terms. Has to be − not +, as in Eq (3), cause you are maximizing not minimizing.

Response: Thank you for catching this - it has been corrected.

10) page 6, Algorithm 1: How do you initialize Ωˆk for the non-convex GMM problem?

Response: Thank you for pointing out this lack of relevant information. We added text to section 2.2.2 describing the details of initialization, and reference this from the paragraph Summary of EM algorithm, on page 9, showing the consistent initialization scheme between the sequential and simultaneous methods:

“We fit the GMM with the \texttt{GaussianMixture} class from \texttt{scikit-learn} \cite{pedregosa_scikit-learn:_2011}, enforcing diagonal covariance matrices (\texttt{covariance_type=`diag'}), initializing the optimizer with the solution of K-means clustering (\texttt{init\_params=`kmeans'}), and choosing the best optimization of those obtained from 20 random initializations (\texttt{n_init=20}).”

11) page 7, ~middle: As already stated, please plot β^stim as additional panels in Fig S3 to support that you can indeed “assume that β^stim_i and β^0_i are independent of the cell type k”.

Response: As discussed in response to the earlier comment, these panels have been added as Figure S3E, with discussion of its relevance for the differences between cases A and B. We left this part of page 7 unchanged, as it already references the appropriate supplement section.

12) page 7, before Eq (11): Strictly speaking, spiking activity of each neuron is independent conditioned on the stimulus.

Response: Thank you for catching this - it has been clarified.

13) page 8, Fig 1B: Wrong symbols for spiking response (x should be y) and stimulus (s should be x).

Response: Thank you for catching this - it has been corrected.

14) page 8, E-step: What was the reason to use the Laplace approximation with diagonal covariances? Wouldn't mean field variational inference, as e.g. Blei et al. do for the GMM in "Variational Inference: A review for Statisticians", yield a better approximation?

Response: We added a note here about why we chose the Laplace approximation, allowing for the possible use of other approximations such as in the suggested citation, and tweaked the paragraph in the Discussion about this to better match the reasons therein. The added note:

"We note that many other approximations may be suitable here, such as mean field variational inference \cite{blei_variational_2017}, but have chosen this one for its simplicity and the low computational cost of the resulting algorithm (see Section \ref{sec:complexity} for details)."

The referenced Complexity section is new, and goes into more detail on this subject and generally the computational complexity of algorithm 2.

15) page 10, Alg 2: Shouldn't the update for $c^{-1}_{i,k}$ be $(\text{diag}(\nabla^2 \log P)^{-1})^{-1}$, as in the supplementary material? Diagonalize the covariance, not the precision matrix?

Response: This was actually a typo in the supplement. We chose to diagonalize the precision matrix and not the covariance because it is computationally faster (no matrix inverse required). This has now been corrected to match the details in the main text.

16) page 10, Algorithm 2: How do you initialize Ω^k for the non-convex GMM problem?

Response: We added text to the paragraph Summary of EM algorithm, on page 9, referencing the explanation for GMM initialization in the sequential method:

"We perform this algorithm 20 times from different random initializations and use the results from the optimization with the lowest loss (see Section \ref{sec:mod_sel}). This choice mirrors that used for the sequential method (see Section \ref{sec:cluster-model}). We also note that the initialization of the cluster parameters on line \ref{line:GMM-init} uses the same settings as for the sequential method, except that only 10 random initializations are used."

17) page 12, section 3.1: typo, 10−5/6 should be 10−5/6

Response: Thank you for catching this - it has been corrected.

18) page 13, Fig 2F: Is there an intuitive explanation why $\sqrt{\Sigma}$ saturates for small σ?

Response: Thank you for asking for this - we believe there is an intuitive explanation that ties in with the principle of borrowing strength. In short, when true sigma is low, the learned sigma (eq S8) is dominated by the posterior widths of individual cells (c_ik); when true sigma is large, the learned sigma is dominated by the between-cell spread in posterior means (m_ik m_ik^T-mu_k mu_k^T, the rest of the argument of diag in eq S8). We added the following text to section 3.1 about the particular value to which it saturates:

“Note that as $\sigma\rightarrow 0$, the estimated cluster spread saturates, reflecting the width of the posterior distribution of the clustered GLM parameters $\boldsymbol{\beta}^\mathrm{self}_i$. This saturating value is about $\sqrt{40}$ times lower for the simultaneous method because it essentially pools the data across all 40 simulated neurons in each cluster to estimate the posterior for a single one.”

19) page 14, Fig 3: Is there an intuitive explanation why simultaneous model underestimates whereas sequential model overestimates K?

Response: If we think of both simultaneous and sequential methods as regularization on GLM parameters, then we can see that the simultaneous method attempts to push these parameters into cluster distributions with a set of shared parameters which would tend to provide a "probabilistic bonus" towards parsimonious clusterings, whereas the sequential method must attempt to cluster parameters that have been fit individually and are high dimensional which might lead to outliers providing excess weight to greater numbers of clusters.

20) page 14, right before section 3.2.1: (weights 0.6-0.12) should be (0.06-0.13) if the legend in Fig 4 is correct.

Response: Thank you for catching this - it has been corrected.

21) page 15, Fig 4: According to panel C, the correct K for the sequential method is K = 19. How would panel D look without forced merging of clusters, i.e. for K = 19?

Response: The clusters for K=19 are shown below. We believe that this tells the same story - that the sequential method finds clusters that are bunched near the origin and highly overlapping. We used K=12 in the main because we felt it made a fairer comparison, as a model with more clusters will appear to have fitted clusters that are more bunched together.

We’ve added this detail in the new supplementary section S6.

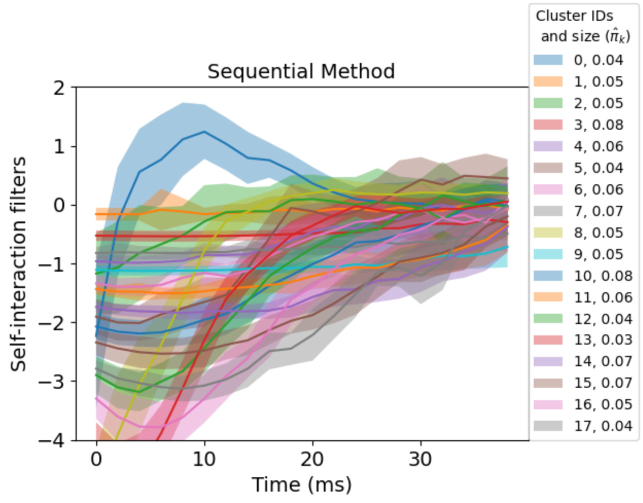

22) page 16, section 3.2.2, 2nd paragraph: “Many metadata labels belonged to certain clusters discovered by the sequential method...” ⇒ “Many metadata labels belonged to certain clusters discovered by the simultaneous method...”

    Response: Thank you for catching this - it has been corrected.

23) Some references are now available not only as preprints but as (hopefully improved) peer-reviewed articles.

    page 21: ref 1 has been published in Nature Neuroscience, ref 13 in Neural Computation.

    page 22: ref 18 has been published in NeurIPS (I know, not really a journal), ref 19 in Cell, and ref 26 in Nature Neuroscience.

    Response: Thank you for catching these - we have updated the references.

24) GitHub: I applaud you for making the code to reproduce the analysis available. However, some scripts won’t run through, e.g. N in data grabber.py is not defined yielding a NameError.

    Please add a license to the repo, otherwise (see https://choosealicense.com/no-permission/) only you as creator are permitted to use the software.

Response: Thank you for taking the time to test our code, and we apologize that it was not yet ready for this test. We have undertaken to refactor our code and generally make it more readable, and have now tested it more thoroughly.

Thank you for the note about a license - we have added a license as license.txt in the repo.

Reviewer #2: The authors propose a hierarchical statistical model, building on generalized linear point process models for single-neuron recordings, to identify functional cell type clusters in neural data. The paper demonstrates the utility and practicality of a hierarchical model to directly probe hypotheses of clustered functional types within a single step, rather than separate GLM and clustering models. The presentation is great: a tutorial-like to guide that could walk a broad audience in the neuro community through the methods in addition to the motivations and results. This manuscript was enjoyable to read and it would make a good addition to PloS-CB pretty much as is. I have included a few suggestions that the authors may wish to incorporate into their final version.

Response: Thank you for the kind words and thoughtful review.

Comments

1) What was the total computation time to fit each approach for each example? This could depend a lot on the size of the grid of penalty terms (\lamdba^{stim} and \lambda^{self}) considered.

   Response: We thank the reviewer for pointing out that this important information was missing. We have created a new Section S1.3 that summarizes the computational complexity of our EM algorithm, the ranges of hyperparameters used and resulting number of optimizations, the hardware system we used for optimizations, and the resulting runtimes.

2) Figure 2B shows that the sequential method primarily fails to fit the larger negative refractory period terms. Additional interpretation here would be helpful. It appears that the ridge penalty is too strong for these large magnitude terms when applied to single neurons, each with independent penalty terms. Pointing out this over-shrinkage effect would provide a nice contrast to the high variance of the sequential method in the next section (Figure 4D).

   Response: Thank you for pointing out this gap in our presentation of results. We’ve added some text to section 3.1 explaining why this happens, and also why it is an effect

that we do not want to affect our analysis in Figure 2E. We added a reference to sec 2.5, where we describe how we avoid this by excluding coefficients less than -4 from RMS measures of parameter accuracy.

“Because only the sequential method applies ℓ_2 regularization to self-interaction filters, those estimates particularly are biased towards 0 (for example, see Figure \ref{fig:fig_sim}B), especially for very negative filter coefficients. However, the exact magnitude of such negative coefficients does not affect the GLM's spiking likelihood much, so we exclude these coefficient estimates from measures of self-interaction filter accuracy (see Section \ref{sec:eval_mets} for details).”

3) Page 7, after equation 9, states “This hierarchical model has two hyperparameters, K, \lambda^{stim}”. What about the spike history penalty term, \lambda^{self}?

   Response: Thank you for asking about this. We have added a more explicit comparison of the prior used for estimating beta^stim_i between the simultaneous and sequential methods here that specifically addresses the difference in hyperparameters:

   “Note that there is no λ^self hyperparameter for ℓ_2 regularization of $\boldsymbol{\beta}^\mathrm{self}_i$ with a Gaussian prior, as the corresponding prior here is the learned cluster distribution (a Gaussian mixture).”

Reviewer #3: The authors present a method to estimate functional cell types by fitting a generative model that simultaneously estimates neurons' cluster identities and the functional (GLM) parameters per neuron (that depend on their cluster). Their method generally outperforms a sequential approach where GLMs are first fit per neuron and then those GLM coefficients are clustered.

I found the paper to be thoroughly written and I only have minor comments.

Response: Thank you for the kind review. We respond in detail to each comment below.

1) I found equation 2 hard to follow. I think it could be useful to have a supplemental figure demonstrating how the covariates are constructed and fed into the GLM.

   Response: Thank you for this feedback. We edited figure 1A to illustrate d^stim (which we are assuming was the confusing element, please let us know if not), and added some intuition-building comments to the descriptions of the various terms in eq 2 in the bullet-points that follow.

2) I would clarify why you are clustering just based on the self-interaction filters in the main text. The results clearly are better when using only self-interaction filters - why do you think this is the case?

   Response: Thank you for this feedback, we agree that this is an important consideration we had not properly addressed, and so did reviewer #1. To avoid redundancy, we refer to our response to that reviewer's first comment. We believe that this specific question is addressed in the second new paragraph added to Section S2.

3) I found the description of EV_ratio in the Methods hard to follow - how does an average (either the model average or PSTH average, which is staying the same) covary with individual trial responses?

   Response: Thank you for pointing out this ambiguity - the covariance we refer to is across time points, not trials. This has now been specified in the text.

4) For figs 1-5, please put the panel letter in the upper left (which is what I always see) instead of lower left. I kept getting thrown off when looking at figures.

   Response: We thank the referee for this comment and agree completely - the panel letters have been moved.

5) It's hard to judge how realistic the simulated data is. To demonstrate that the method works on simulated data that is similar to neural data, I would recommend the following additional approach: 1) Fit the model to your actual data - this model will be your ground truth model; 2) Generate simulated data from that ground truth model (which should hopefully be fairly similar to the true neural data); 3) Fit a new model to the simulated data and see how well it can recover the ground truth parameters.

   Response: This is an important concern that we thank this reviewer for raising. To begin, we must highlight a general problem with simulating spike trains from Poisson GLMs that constrains any simulation study, including others in the literature (e.g. Weber et al. 2017). We have added the following to our explanation of our simulation setup in Section S4:

   "When $\sigma>10^{-5/6}$ is used, the simulation becomes unstable, because some $\boldsymbol{\beta}^\mathrm{self}_i(\tau)$ values become high enough to cause runaway feedback, whereby the number of spikes in bins spaced τ apart diverge to infinity with increasing time. This critical value of $\boldsymbol{\beta}^\mathrm{self}_i(\tau)$ is around $-[\beta^0+\max_t x_i(t)\sum_{t=1}^{T^\mathrm{stim}}\boldsymbol{\beta}^\mathrm{stim}_i(t)]$, such that a single spike τ time bins ago can raise the spike rate above 1 if it occurs during an

extended period of high stimulus values. To prevent this scenario, we impose $y(t)\in\{0,1\}$ while simulating (as in \cite{weber_capturing_2017}), and do not consider such high values of σ that would lead to such severe model misspecification that the true model parameters cannot be recovered. It is worth noting that this problem can arise even for filters fitted to real neural data because of model misspecification - specifically, there is a maximum number of times any neuron can spike in a given time bin because of its absolute refractory period, whereas the (Poisson) GLM makes no such assumption."

To show that this problem does indeed arise from the parameters fitted in this dataset, below We've included a heatmap of the differences between the maximum self-interaction filter coefficients and their corresponding critical values (x-axis) of GLMs individually fitted to recorded neurons (sequential method, left panel) and of those obtained from the simultaneous method (right panel). When this critical difference is much greater than 0, one would expect that unstable simulations are likely, and you can see that this is indeed the case, as measured by the number of times the simulated rate exceeds 1: when the critical value is near 0, there is a sharp transition.

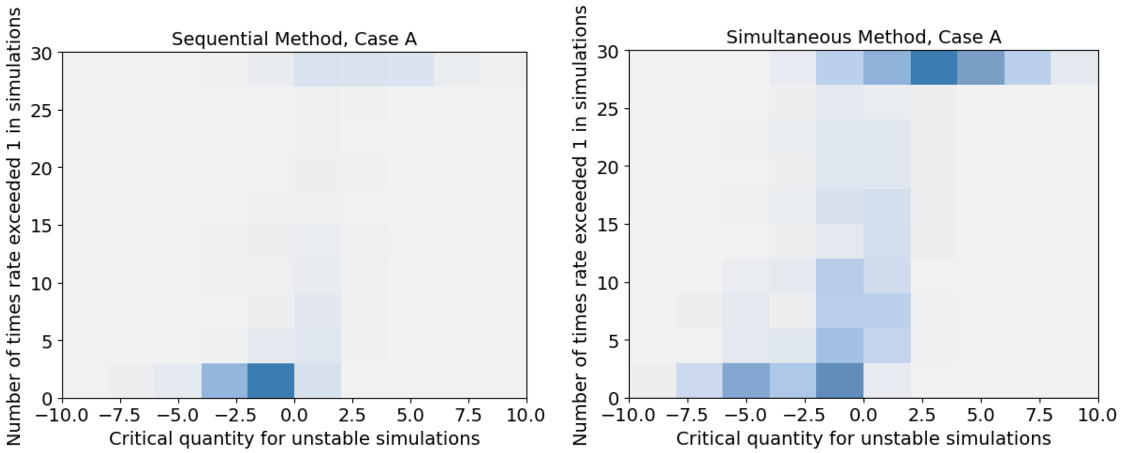

Because of this reality, the approach suggested would fail when applied to parameters fitted to an entire dataset, so we apply it only to neurons from the five clusters with the lowest critical values, above a threshold of -2 (below this, the simulated neurons produce very few spikes). Specifically, we use the 95% confidence interval of critical values for each cluster using the fitted mean and variance, and check that the bottom is not below -2, and then choose the five with the lowest upper edge of the confidence interval. We then sample single-cell parameters from this five-cluster distribution and generate simulated spike trains. We do this only for the Case B model because it is more straightforward to determine how all the single-cell parameters should be sampled. The results are shown in the new supplementary section S4.1.

6) For the line before section 3.2.1: I believe 0.6 should be 0.06

Response: Thank you for catching this - it has been corrected.

7) I'm confused by the following statement: "Given that different populations of neurons are responsible for changes in ANLL and EVratio, it is no surprise that these metrics scale differently with the number of presentations used for training." Why does the number of training examples affect different populations of neurons (high and low firing) differently?

   Response: The question of why the different metrics scale differently with number of stimulus presentations is interesting, and we thank this reviewer for probing deeper into it. While we believe that a more thorough answer to this question would require further analysis that is beyond the scope of this paper, we expounded upon our reasoning for how the role of different neural populations may result in different scaling for each metric, providing a concrete example of how this much work:

   "One potential reason for this is that one of these populations may have a less variable response to the repeated stimulus, yielding a narrower posterior over GLM parameters, than the other."

8) In section 3.2.2, could you please expand upon (or make more concrete) the following sentences: "...tend to have similar correlations with specific attributes (especially dendrite type). This suggests that these attributes may not be linked to cell type per se, but rather to an electrophysiological feature that varies continuously between cell types." This result seems to be an important practical difference between the simultaneous and sequential approaches, and it can be challenging to understand upon the first read.

   Response: We are very grateful for this comment - it has pushed us to attend more closely to the relationship between cell types discovered in a functional space and discrete attributes imposed on continuous measurements of cell morphology, location, and transgenic expression. We have rewritten much of the referenced paragraph, and believe its message is now both clearer and more meaningful. In short, the smooth horizontal and vertical gradients that appear in many panels make sense if the discovered cell types and provided attributes represent well-ordered divisions of a uniform distribution of cells, while the discontinuous transitions make sense if the identified clusters and provided attributes really represent a clustered distribution of cells. The fact that both are observed suggests that the discovered cell types are related to the provided metadata, and that each may represent meaningful divisions of an inherently continuous space.

9) I would mention the github repository in the paper so people know where to find it. I would also strongly recommend that the repository/code is complete and easy-to-follow (e.g. commented) prior to acceptance (so others can easily use and adapt your method).

   Response: We thank the reviewer for this input - we added a link to the repository at the top of the methods section. We have endeavored to make the code more accessible,

and will continue to do so as we add to it in addressing these reviews, prior to acceptance.
